# Engineering neurovascular thrombosis: Light-based bioprinting for patient-specific modeling and women’s cerebrovascular health

**DOI:** 10.1126/sciadv.aeh3952

**Published:** 2026-10-02

**Authors:** Linda Dei-Awuku, Nicole A. Yap, Yunduo Charles Zhao, Zihao Wang, Mary M. Kavurma, Jiawen Li, Xiaoxue Xu, Jennifer Quon, Yan Yan S. Huang, Khoon S. Lim, Kim-Lien Nguyen, Tzung K. Hsiai, Lining Arnold Ju

**Affiliations:** ^1^ School of Biomedical Engineering, The University of Sydney, Darlington, NSW 2008, Australia.; ^2^ Charles Perkins Centre, The University of Sydney, Camperdown, NSW 2006, Australia.; ^3^ The University of Sydney Nano Institute (Sydney Nano), The University of Sydney, Camperdown, NSW 2006, Australia.; ^4^ Heart Research Institute, Camperdown, Newtown, NSW 2042, Australia.; ^5^ Centre for Peripheral Artery Disease, Heart Research Institute, Newtown, NSW 2042, Australia.; ^6^ School of Medical Sciences, The University of Sydney, Camperdown, NSW 2006, Australia.; ^7^ School of Electrical and Mechanical Engineering, Institute for Photonics and Advanced Sensing (IPAS), Adelaide University, Adelaide, SA 5000, Australia.; ^8^ School of Biomedical Engineering, University of Technology Sydney, Ultimo, NSW 2007, Australia.; ^9^ Department of Surgery, University of Toronto; Division of Neurosurgery & SickKids Research Institute, The Hospital for Sick Children, Toronto M5G 1X8, Canada.; ^10^ Department of Engineering, University of Cambridge, Cambridge CB2 1PZ, UK.; ^11^ The Nanoscience Centre, University of Cambridge, Cambridge CB3 0FF, UK.; ^12^ Sydney Biomanufacturing Incubator, The University of Sydney, Camperdown, NSW 2006, Australia.; ^13^ Department of Medicine, David Geffen School of Medicine at UCLA, Los Angeles, CA 90095, USA.; ^14^ Department of Bioengineering, University of California, Los Angeles, CA 90095, USA.; ^15^ Department of Radiological Sciences, David Geffen School of Medicine at UCLA, Los Angeles, CA 90095, USA.

## Abstract

Cerebral venous sinus thrombosis (CVST) and carotid web (CW) are distinct cerebrovascular disorders where vascular geometry, disturbed hemodynamics, and endothelial activation interact to drive thrombosis. Both conditions disproportionately affect women, yet remain understudied because of the inability of traditional platforms to replicate complex three-dimensional lumen profiles. This perspective positions light-based lithography as a powerful biofabrication strategy for patient-specific modeling. Digital light processing and volumetric bioprinting render enclosed, perfusable hydrogel networks with high-fidelity surface topography. When integrated into dynamic vessel-on-chip perfusion loops guided by clinical imaging and computational fluid dynamics, these platforms simulate pathological flow stasis and recirculation zones, heterogeneous shear-stress gradients, and site-specific cellular aggregation. This biofabrication approach enables personalized disease modeling and translationally relevant risk assessment, providing a critical platform for advancing women’s cerebrovascular health.

## INTRODUCTION

Cerebrovascular diseases remain among the leading causes of death and disability worldwide, accounting for immense clinical and socioeconomic burden (*1*, *2*). While much attention has centered on arterial pathologies such as ischemic stroke, aneurysms, and arteriovenous malformations, disorders of the cerebral venous and carotid vasculature have received comparatively little focus. Cerebral venous sinus thrombosis (CVST), though rare, is one of the most devastating forms of venous stroke (*3*). It represents approximately 1% of all strokes (*4*), yet it carries a substantial clinical burden given its often sudden onset, diagnostic difficulty, and long-term sequelae, including intracranial hypertension, seizures, and cognitive impairment. Mortality remains around 5 to 10% in acute stages, and up to one-third of survivors experience lasting neurological deficits (*5*).

Recent studies have further highlighted the evolving clinical landscape of CVST in the context of the coronavirus disease 2019 (COVID-19) pandemic (*6*). Population-level and cohort studies have reported an increase in CVST incidence during pandemic periods, alongside a strong association between severe acute respiratory syndrome coronavirus 2 (SARS-CoV-2) infection and thrombotic complications driven by systemic hypercoagulability and endothelial dysfunction (*7*).

Notably, CVST presents a disproportionate prevalence in women of reproductive age (*8*). This profound sex disparity suggests that the female lifecycle, which encompasses puberty, pregnancy, and menopause, exposes the vascular endothelium to dynamic hormonal fluctuations that alter vascular tone, vessel compliance, and prothrombotic susceptibility (*9*). Estrogen substantially modulates vasodilator pathways, nitric oxide production, and vascular smooth muscle cell (SMC) proliferation; consequently, fluctuations in hormone levels may modulate endothelial and vascular responses to disturbed flow, potentially influencing susceptibility to thrombosis in geometrically complex venous environments.

Another underrepresented neurovascular condition is the carotid web (CW). Despite its arterial origin, both the biological basis and clinical understanding of this condition remain limited. CW has been identified to account for up to 9.58% of cryptogenic stroke cases (*10*), and 13% prevalence in young patients with stroke. With most clinical attention focused on more common etiologies, patients with CW often go misdiagnosed or classified as cryptogenic. This is especially problematic as CWs primarily affect younger, female patients of African heritage who otherwise have prime cardiovascular health (*11*). The largely disproportionate populations of risk and the lack of recognition across healthcare fields represent an important area to address, especially given the hemorrhagic risks associated with general antithrombotic therapies administered to these patients and the low number of targeted studies and access to specialist healthcare services for African populations.

CVST and CW represent distinct vascular territories and serve as representative yet understudied cases that are unified by a shared reliance on complex, nonlinear fluid dynamics. In both pathologies, the initiation of disease is fundamentally governed by geometric predisposition, where the architecture of the multiaxial dural sinus junctions and the intraluminal carotid shelf dictate the local mechanical environment (*12*). A major barrier to studying these structures is the lack of physiologically relevant in vitro models. Conventional vascular chips typically rely on simplified, planar microchannels or generic arterial constructs (*13*) that fail to recapitulate complex junctional angulations, low-pressure recirculating flows, and highly variable patient-specific anatomy (*4*, *14*). Consequently, existing models fall short in elucidating these specific stroke mechanisms or supporting translational drug and hormone testing (*15*).

In this perspective, we highlight advanced light-based bioprinting and microfluidic platforms as promising approaches for these anatomical and sex-specific modeling gaps. Technologies such as digital light processing (DLP) (*16*) and volumetric bioprinting (VBP) (*17*) facilitate the fabrication of fully enclosed, monolithic vascular structures with sub–100 μm resolution. This precision is a functional requirement for capturing the high-fidelity mechanobiological drivers of CVST and CW, such as sharp intimal shelves and asymmetric dural junctions, where surface topography directly dictates the activation of sensitive hemodynamic sensors. Unlike existing vascular-on-chip platforms that rely on simplified cylindrical or planar geometries, the proposed framework leverages light-based bioprinting to achieve patient-specific anatomical fidelity at sub–100 μm resolution, capturing the tortuous three-dimensional lumen profiles and sex-specific mechanobiological drivers that are absent from conventional models.

By integrating machine-learning-based segmentation with the seamless surface fidelity of DLP, VBP, and microfluidics, the proposed approach moves beyond generic vascular constructs toward sex-specific systems. These platforms enable a more rigorous assessment of the distinct vascular morphology and hormonally mediated endothelial responses unique to women’s cerebrovascular health, providing a robust foundation for personalized risk assessment and preclinical drug testing.

Figure 1 provides a simplified schematic overview of the disproportionate epidemiological profile of CVST and CW, along with the anatomical locations of the carotid artery and cerebral venous sinus within the cerebrovasculature. The labeled vessel branches represent areas within the cerebral artery and venous system where thrombosis due to CW and CVST are most likely to occur, respectively. Enlarged windows of the carotid artery bifurcation and the highly branched transverse sinus show examples of where CW and CVST pathological thrombosis is likely to occur due to the complex geometries associated with these conditions. Diagnosis of CVST via imaging can be performed efficiently, however the time-variable behavior of the clot and nonspecific symptoms often delay diagnosis. On the other hand, identification of the CW is often overlooked or misdiagnosed on imaging due to the lack of clinical recognition and routine imaging procedures unsuitable for reliable diagnosis of the web. Because of the thin flap morphology of the CW, thick computed tomography slices and magnetic resonance imaging (MRI) techniques used commonly for stroke imaging often miss the structure, while digital subtraction angiography (DSA) is not commonly used because of the invasive nature of the imaging modality (*18*). In addition, the CW is often mischaracterized as atherosclerotic plaque, local thrombi, or arterial dissection. These factors lead to many indeterminate causes of stroke amongst women and younger patients.

**Fig. 1.**
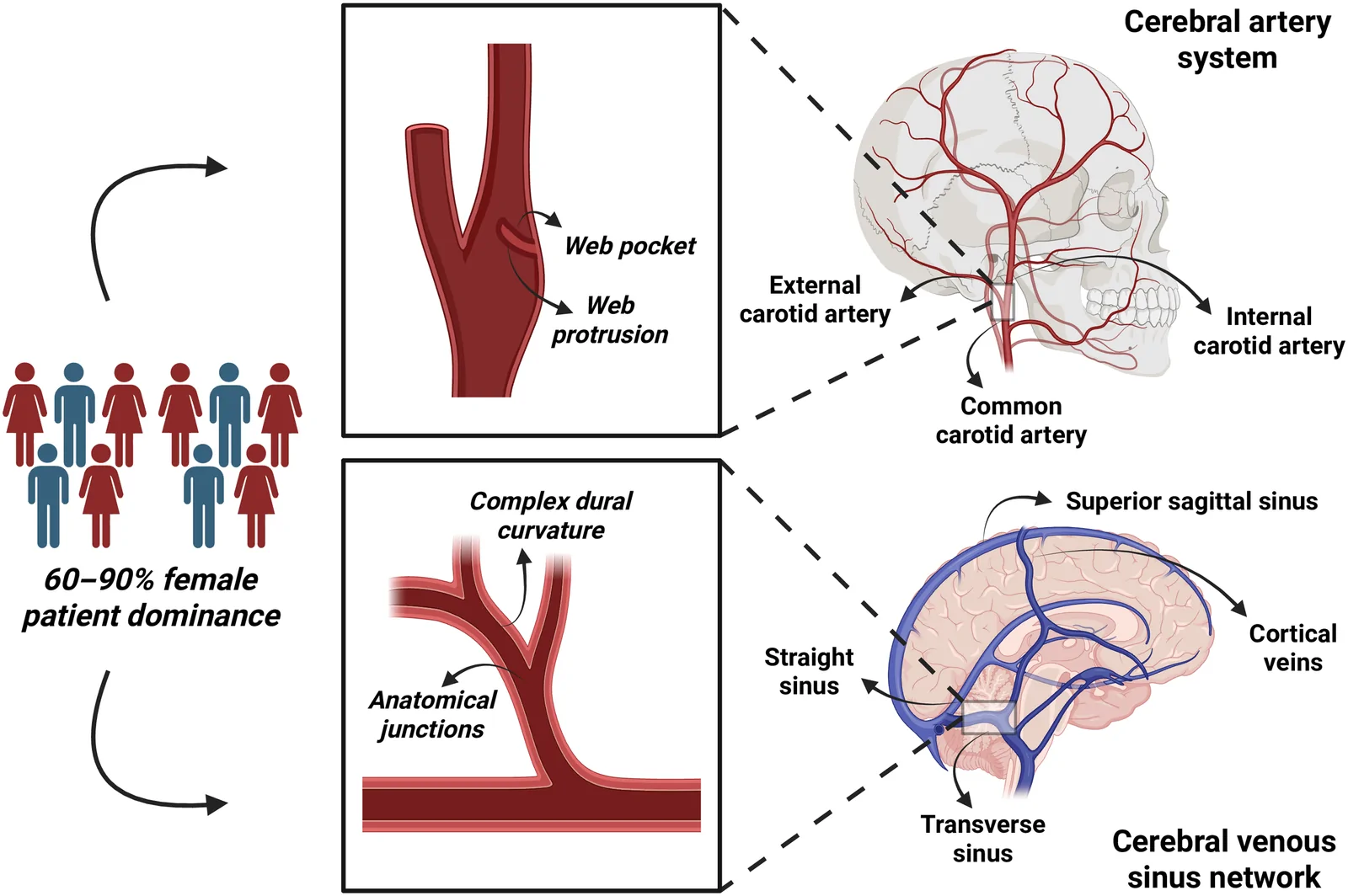
The disproportionate burden on females and the anatomy of CVST and CW. Labeled vessels represent the regions of high thrombotic risk and stroke burden. (Top window) Web pocket and protrusion of the CW are thrombosis-prone regions. (Bottom window) Complex dural curvature and anatomical junctions of the CVS are thrombosis-prone regions. [Created in BioRender. Yap, N.A. (2026) https://BioRender.com/hd2748j].

Here, we aim to provide a comprehensive and structured approach to modeling CVST and CW, capturing their minute yet important geometrical details, multicellular vascular compositions, complex hemodynamic flow, and sex-specific hormonal influence. The approach would allow researchers to link disease-specific mechanisms to classic endothelial cell (EC) health and mechanosensitive markers, inflammatory markers, and thrombotic end points. It represents a critical foundation for interrogating similarly unique cerebrovascular phenomena that remain poorly managed in clinical settings and elusive in etiology, especially given the heterogeneous presentations as seen for CVST. Through personalized bioprinted modeling, we may better understand the disproportionate risk of CVST and CW in women, design more pathologically informed interventions, perform cohort triage, and use the platforms for personalized disease risk and treatment evaluations.

## CVST: A COMPLEX AND DISTINCT NEUROVASCULAR DISORDER

### Anatomical and hemodynamic complexity of cerebral venous sinuses

The cerebral venous sinuses are dura-lined endothelial channels that drain venous blood from cortical and deep cerebral veins into the internal jugular system. Unlike arteries, cerebral venous sinuses are nonmuscular, dura-lined endothelial channels that lack smooth muscle layers and elastic laminae, rendering them highly compliant and incapable of active vasoregulation (*19*). Their architecture is characterized by extreme interindividual variability, exhibiting marked cross-sectional asymmetry, tortuous curvatures, and intrinsic lumen obstructions from arachnoid granulations and fibrous septa. Operating under low-pressure, low-velocity, and minimally pulsatile conditions, this network is highly sensitive to geometric variation (*20*). Crucial macro-anatomical junctions, such as the torcular Herophili and lateral sinus bifurcations, act as natural stagnation points that prolong blood residence times independently of systemic drivers. Consequently, acute CVST progression is uniquely dictated by this spatial topology; mechanical outflow obstruction drives a rapid rise in upstream intracranial pressure and parenchymal edema rather than intrinsic vascular remodeling (*21*).

### Clinical imaging and disease characterization

Clinical understanding of CVST has traditionally been derived from neuroimaging modalities such as MRI, magnetic resonance venography (MRV), and susceptibility weighted imaging, which enable visualization of venous occlusion, congestion, and secondary parenchymal injury (*22*). Conventional contrast-enhanced techniques, however, often exhibit limited sensitivity in low-flow venous systems and incomplete delineation of thrombus morphology and sinus lumen geometry. Advanced contrast agents such as ferumoxytol (*23*), ultrasmall superparamagnetic iron oxide nanoparticles with prolonged intravascular residence time, have demonstrated enhanced sensitivity for imaging cerebral venous sinuses and thrombus burden, enabling improved visualization of venous architecture, flow impairment, and thrombotic composition (*24*). Such imaging approaches provide higher-fidelity anatomical and pathological characterization of CVST, thereby strengthening patient-specific disease mapping and mechanistic understanding. These advances in venous imaging directly support the development of biomimetic cerebrovascular models by improving the accuracy of anatomical segmentation, geometric reconstruction, and hemodynamic parameterization, thereby linking clinical diagnosis to experimental model design.

Among available imaging modalities, MRI offers a detailed assessment of venous occlusion, congestion, and secondary parenchymal injury. However, interpretation can be challenging because thrombus signal characteristics vary throughout the course of thrombus evolution; very acute thrombi may mimic normal flow voids, whereas chronic or partially recanalized lesions may be less conspicuous on conventional imaging sequences (*25*).

### Disproportionate risk in women

One of the most defining epidemiological features of CVST is its strong female predominance. Seventy to 80% of all CVST cases occur in women of reproductive age, a pattern remarkably consistent across regions and ethnic groups (*8*). This represents one of the most pronounced sex disparities among cerebrovascular disorders and underscores the biological distinctiveness of CVST within the broader stroke spectrum (*26*). The most extensively documented contributor to this disorder is hormonal contraception, particularly the combined estrogen-progestin formulations. Large cohort studies have demonstrated that oral contraceptive use increases the risk of CVST by approximately five- to 20-fold, depending on formulation, dosage, and duration of use. While physiological hormonal fluctuations during the regular menstrual cycle may modestly influence venous thrombotic risk, the incidence of CVST increases substantially during pregnancy and the puerperium, with reported three- to 10-fold higher risk in the postpartum phase. Pregnancy induces a natural hypercoagulable state through elevated levels of fibrinogen, factor VIII, and von Willebrand factor (vWF), coupled with reduced fibrinolytic activity (*9*). These systemic changes, combined with venous stasis and dehydration during labor or the early postpartum period, create a transient but potent prothrombotic environment. Anatomical adaptations, such as venous dilation and endothelial stress, further compound the risk, particularly within the dural sinuses, where low-shear zones are predisposed to clot formation (*27*).

Beyond these physiological factors, hormone replacement therapy and assisted reproductive technologies contribute to emerging risk profiles. Estrogen supplements used in menopausal management and fertility treatments exert similar hemostatic effects, elevating circulating coagulation factors and suppressing anticoagulant pathways (*28*). Case reports and small series have documented CVST events following ovarian hyperstimulation or exogenous hormonal cycles, suggesting that iatrogenic endocrine modulation may play a larger role than currently recognized (*29*). These risks do not occur in isolation but within a broader context of sex-specific vascular physiology. Estrogen modulates endothelial nitric oxide production, vascular tone, and platelet reactivity; its withdrawal or exogenous fluctuation may destabilize endothelial homeostasis (*30*). Venous endothelium expresses estrogen and progesterone receptors, suggesting that hormonal states can directly influence endothelial gene expression and the local balance between pro- and antithrombotic signaling (*31*). This mechanobiological interface between hormone exposure and venous hemodynamics may explain why certain individuals develop CVST despite minimal systemic hypercoagulability.

Diagnostic delays are common, with women often experiencing nonspecific early symptoms such as headache or visual disturbance that are dismissed as benign (*32*). The average time from symptom onset to confirmed diagnosis can exceed several months to years, contributing to preventable morbidity. Understanding its disproportionate impact on women requires an integrated approach that combines mechanistic vascular modeling, hormonal profiling, and equitable clinical awareness. Table 1 highlights the hormonal and reproductive risk factors associated with CVST in women.

**Table 1.** Hormonal and reproductive risk factors for CVST in women.

Risk factor	Hormonal mechanism	Estimated CVST risk	Key hemostatic effects	Ref.
Combined oral contraceptives	Exogenous estrogen-progestin exposure	5–20× baseline	↑ Coagulation factors (VII, X, fibrinogen); ↓ protein S; impaired fibrinolysis	(*121*)
Pregnancy	Physiological hypercoagulability	3–10× (highest postpartum)	↑ Fibrinogen, factor VIII, vWF; venous stasis; dehydration during labor	(*8*)
Hormone replacement therapy	Exogenous estrogen (menopausal management)	Elevated; dose-dependent	↑ Circulating coagulation factors; suppressed anticoagulant pathways	(*122*)
Assisted reproductive technology	Ovarian hyperstimulation; exogenous hormonal cycles	Case-level; poorly quantified	↑ estrogen and procoagulant factors during ovarian stimulation	(*123*)

## CW AND SHARED MECHANISMS IN CEREBROVASCULAR THROMBOSIS

### Introduction and epidemiology of CWs

The CW is another underrepresented cerebrovascular anomaly. Figure 2 depicts the structure of the CW as a flap extending into the carotid artery lumen of a patient, obtained through catheter angiography. Despite increasing recognition as a potential cause of ischemic stroke, the biological origins, pathophysiological mechanisms, and thrombogenic potential of CW remain incompletely understood. Because of the lower prevalence of cases among patients with stroke, CW has been largely sidelined by clinical and research communities, and often misdiagnosed as cryptogenic or stroke of other etiologies. Cryptogenic stroke refers to strokes of unknown etiology, where patients present with no identifiable risk factors such as atherosclerosis, arterial dissection, deep vein thrombosis, or atrial fibrillation. CW features have been identified in ∼13% of younger patients with stroke (*33*), typically occurring in otherwise healthy individuals. While CW prevalence remains low, 1.2 to 2.5% across stroke patient databases in the United States and the Netherlands (*34*), emerging evidence suggests that CW is disproportionately represented among patients with cryptogenic stroke. In these populations, reported prevalence ranges from 6.4% (*35*) to 9.58% (*36*), corresponding to odds ratios of 10.1 to 12.5 compared with control populations (*37*).

**Fig. 2.**
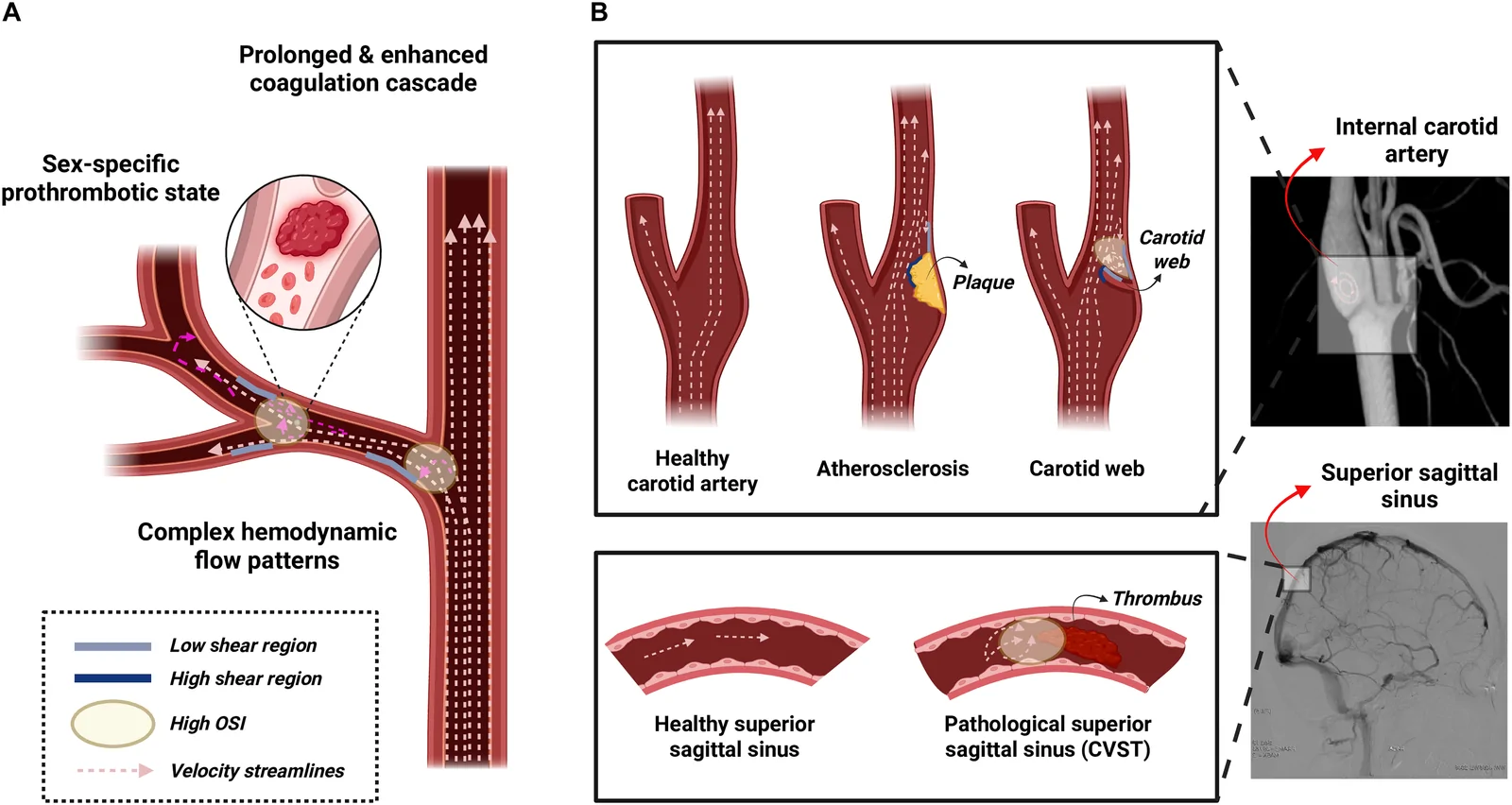
The altered hemodynamics of CVST and CW. (**A**) The interplay of complex hemodynamic patterns and sex-specific female hormone influenced prothrombotic state leads to the characteristic thrombotic etiology of CVST and CW under a prolonged coagulation cascade in low shear recirculating flow. (**B**) (Top) The anatomical location on DSA and flow patterns in the carotid artery bifurcation showing healthy laminar flow, atherosclerotic high shear flow, and CW variable shear flow with a recirculation zone posterior to the flap. (Bottom) The anatomical location on DSA and flow patterns in the cerebral superior sagittal sinus show healthy laminar flow and pathological recirculating flow due to thromboembolic events. [Created in BioRender. Yap, N.A. (2026) https://BioRender.com/kcfe2do. Anatomical diagrams adapted with permission from (*117*) (https://neuroangio.org/) and from (*118*)].

However, despite its relatively low prevalence, the risk of stroke recurrence among patients with CW is disproportionately high. Reports have shown ipsilateral recurrence rates ranging from 24 to 71.4% within a median of 9 to 13 months (*38*). Importantly, CWs disproportionately affect younger patients of cryptogenic stroke, with ∼27% experiencing a recurrent attack within 5 years. Beyond its higher prevalence in otherwise healthy and young patients (<60 years) (*10*), CW is closely associated with female sex and African ancestry. Large cohort studies and literature report female dominance ranging from 60.9 to 91% (*34*), with one study reporting that 82% of affected women had no external cardiovascular risk factors. In the largest U.S. study to date, 50% of patients with CW were of African heritage (*39*) with a mean age of 63 years, highlighting a disproportionately high relative risk in African Americans within a predominantly Caucasian population. Other studies report African heritage rates ranging from 58 (1285 patients) to 77.8% (681 patients) and median ages of 40.5 to 55 years.

### Disproportionate risk in women

Like CVST, CW shows a marked female predominance, although its underlying sex-specific mechanisms are unclear. Whether CW is congenital or acquired is also unknown, and its prevalence may be underestimated. The absence of CW in pediatric patients with stroke suggests that the condition is not congenital in nature (*40*). As individuals age, vascular tone, remodeling, stiffness, blood pressure, and inflammatory responses diverge between males and females (*41*). Females generally exhibit lower arterial stiffness than males between puberty and menopause, and lower baseline blood pressure and femoral intima-media thickness (*42*). However, these cardiovascular factors increase with age, especially post menopause.

These differences have largely been attributed to estrogen, which exerts cardioprotective effects by stimulating nitrogen oxide production from vascular ECs, keeping blood pressure low, reducing immune cell trafficking and infiltration at vascular wall injuries, and inhibiting growth factor production that is linked to SMC proliferation and intimal-medial thickening in rat models (*43*). Consequently, hypertension and cardiovascular complications, including strokes, are less common in women before 45 years of age, but exceed those of males after 65 years (*44*). This pattern suggests that hormonal fluctuations may underlie the development of CWs, especially given the lack of genetic associations and the rarity in children. Notably, the median age of CWs diagnosis (42 to 56 years) (*45*) overlaps with the menopausal transition, a period characterized by declining estrogen levels and cardiovascular vulnerability (*46*). Ethnic differences further support a hormonal contribution. For example, African women experience earlier menopause (*47*) and prolonged hormonal transition periods as well as higher estrogen levels than other ethnicities (*48*). Together, these observations suggest that both the magnitude and duration of hormonal fluctuations across the lifespan may contribute to the increased prevalence of CWs, particularly among women of African ancestry.

Despite hormonal influences on cardiovascular health, much of the research underpinning current understanding of vascular SMC stiffness and hypertension-associated arterial stiffness in aging has been conducted in males or animal models that do not completely recapitulate the influence of hormones. The reduced effectiveness of antihypertensive medications in women highlights the limitations of these approaches and underscores the need for models that better capture female-specific cerebrovascular mechanisms.

## CURRENT STATE OF THE ART OF VASCULAR MODELS

### Mechanobiology of CVST and CW

Replicating cerebrovascular thrombosis in vitro requires replicating not just the structure of the vessel but the interplay of its geometry, hemodynamics, and endothelial behavior. Virchow’s Triad refers to factors that represent causes of pathological thrombogenesis: altered hemodynamics, endothelial dysfunction, and hypercoagulability. In both CVST and CW, subtle anatomical irregularities create zones of disturbed flow that fulfill these conditions (*49*). Figure 2 illustrates the altered hemodynamic environment contributing to pathological clot formation in CVST and CW.

Figure 2A provides an overview of how complex hemodynamic flow patterns, such as low wall shear stress (WSS) and high oscillatory shear index (OSI) flow in vascular junctions in CVS and carotid bulbs, and spatially heterogeneous and variable WSS at the asymmetric sinus junctions and the CW flap, interact with sex-specific hormonal effects to generate a prothrombotic area. As female hormones induce a natural hypercoagulable state and hormone level fluctuations affect EC function, possibly dysregulating the antithrombotic nature of the vascular wall, the risk of clot coagulation rises, particularly in pockets of low shear and high recirculation characteristic of CVS junctions and CW.

Figure 2B illustrates differences in the hemodynamic environments of healthy and pathological carotid arteries and the superior sagittal venous sinus. Both atherosclerotic plaques and CWs generate altered wall shear profiles and disrupted flow distal to the vessel narrowing. However, while atherosclerotic vessels have a characteristic high shear gradient and generally high arterial flow rates that dictate the shear-induced arterial thrombotic process, CW thrombosis is caused by a large recirculating, low-velocity flow pocket distal to the web. The flow recirculation zone posterior to the flap is considered the primary site of thrombogenesis, consistent with clinical flow contrast images that show stagnation in the flap pocket. Arterial flow contrast images of the CW are best captured via catheter angiography or DSA, as depicted on the right. More recently, iron-based contrast imaging techniques, such as ferumoxytol-enhanced magnetic resonance angiography [(FE)-MRA], have been developed. Ferumoxytol offers the benefits of supplementary iron repletion in women and provides an imaging modality that does not depend on ionizing radiation. Both dynamic and steady-state (FE)-MRA can generate diagnostically valuable 3D images of the CW structure for in vitro modeling (114).

On the other hand, CVST is commonly caused by thrombus formation at low shear, high-OSI pockets within the CVS. The formation of obstructive clots in these areas may lead to obstructed blood flow and perfusion deficits captured best by MRV techniques. These clots further disrupt the venous flow patterns, leading to flow recirculation that reinforces thrombogenesis and thrombus development. Table 2 provides an overview of mechanobiological hallmarks across both conditions, highlighting their implications for in vitro model design.

**Table 2.** Clinical, physiological, and mechanobiological hallmarks of CVST and CW. WSS, wall shear stress; SMC, smooth muscle cells; CEA, carotid endarterectomy.

Feature	CVST	CW	Implications for in vitro modeling	Ref.
Vessel type	Low-pressure dural sinus	High-pressure carotid bifurcation	Different shear regimes are required	(*12*, *124*)
Anatomical structure	Rigid dural sinuses; tortuous, asymmetric with junctional angulations	Intimal shelf projecting into the high-pressure carotid bulb	Patient-specific enclosed geometry; sub–100 μm surface fidelity	(*8*, *34*)
Primary cell sensor	Piezo1/primary cilia	Integrins/vWF conformational sensors	Sensors detect specific flow disturbances	(*125*, *126*)
Key hemodynamic driver	Low-shear stasis; oscillatory flow; recirculation at junctions (WSS <1 Pa)	High local variable WSS; low-velocity recirculation zone in the web pocket	Dual shear-flow capability; venous and arterial flow regimes	(*40*, *50*)
Endothelial response	↓ KLF2/eNOS; ↑ ICAM-1, VCAM-1, TF; procoagulant activation	Intimal hyperplasia and fibroplasia of fibroblasts and SMC, wall thickening	Perfusable endothelialized constructs with real-time imaging capability	(*94*, *127*)
Thrombogenic pathway	Fibrin-dominant “red” thrombus; erythrocyte-laden; mural propagation	Coagulative thrombus in recirculation pocket; embolic detachment to the distal cerebral vessels	Whole-blood perfusion assay; time-to-occlusion and embolic readouts	(*53*, *128*)
Clinical challenges	Nonspecific symptoms	Radiological under-/misdiagnosis	Need for patient-specific constructs to better diagnose CVST and CW	(*3*, *129*)
Treatment	Anticoagulation	CEA/stent	In vitro testing could guide the decision	(*130*, *131*)

#### Venous mechanotransduction in CVST

CVST arises within a structurally complex venous network whose geometry, rigidity, and pressure conditions differ fundamentally from those of arteries. Unlike elastic vessels, the cerebral venous sinuses are rigid, dural-bound conduits that exhibit marked variability in caliber and curvature (*12*). These features, specifically at the asymmetric junctions of the superior sagittal and transverse sinuses, produce spatially heterogeneous WSS and low-shear pockets that act as nucleation sites for clot formation (*50*).

Endothelial dysfunction is the mechanistic link between these disturbed hemodynamics and the onset of thrombosis (*51*). ECs perceive the inherently low WSS (<1 Pa) and high oscillatory shear characteristic of dural sinuses through specialized mechanosensors, including primary cilia and the ion channel Piezo1 (*52*). In stagnation zones created by complex junctions, the lack of laminar flow leads to the localized suppression of KLF2 and eNOS transcription. This molecular failure reduces nitric oxide bioavailability while simultaneously up-regulating ICAM-1, VCAM-1, and tissue factor (TF). TF initiates the extrinsic coagulation cascade, converting the quiescent endothelium into a prothrombotic surface that recruits leukocytes and initiates the fibrin-dense, erythrocyte-laden “red” thrombi characteristic of venous stasis.

Beyond geometry, inflammation acts as a central driver of immunothrombosis, where inflammatory signaling and coagulation are tightly coupled (*53*). Recent vein-on-chip studies have successfully reproduced these effects by subjecting endothelialized sinus geometries to controlled perturbations. For instance, exposure to proinflammatory stimuli induced junctional reorganization and elevated adhesion molecule expression by up to eightfold. When whole blood was perfused at physiological venous shear rates, fibrin deposition propagated preferentially along the inner walls, mirroring clinical observations of mural clot growth (*54*, *55*). The thrombogenic process in CVST evolves within a low-shear environment. Venous WSS in the dural sinuses typically remains below 1 Pa, a regime that promotes fibrin polymerization rather than platelet aggregation. Computational fluid dynamics and patient-specific flow mapping consistently identify recirculation zones and stagnation pockets at anatomical junctions such as the torcular Herophili and sigmoid outlet. These zones exhibit high residence time and oscillatory shear, encouraging local thrombin accumulation and activation of the coagulation cascade. Under these disturbed conditions, the endothelium up-regulates TF and vWF while releasing microparticles rich in procoagulant phospholipids. The result is a fibrin-dense, erythrocyte-laden “red” thrombus that adheres to the sinus wall and propagates longitudinally with the venous flow (*56*). These findings demonstrate that thrombus polarity and stability are directly governed by the synergy between flow-induced mechanosensing and the inflammatory state of the sinus wall.

#### Arterial mechanotransduction in CW

CWs exhibit an arterial condition characterized by larger regions of low WSS, low velocity, high OSI, and flow stasis than in mild atherosclerotic and normal vessels (*57*). ECs perceive these hemodynamic shifts through primary cilia, integrins, and mechanosensitive ion channels such as Piezo1. The primary cellular response in the CW is driven by the extreme shear stress gradients at the base of the intraluminal shelf. As blood accelerates over this protrusion, the mechanical force triggers a conformational change in vWF from its globular inactive state to an elongated state. This elongation substantially increases vWF affinity for platelet GPIbα receptors, facilitating rapid thrombus formation even before substantial endothelial injury occurs. The influence of vWF in arterial systems has been systematically demonstrated on existing vessel-on-chip (VoC) platforms that aim to generate patient-specific physical twins of their in vivo vascular geometry. The study showed that complex flow environments in carotid artery bifurcations led to vWF-mediated thrombotic responses. Given the demonstrable link between vascular geometry and vWF-based platelet mechanotransduction in arterial systems, the complex hemodynamic flow arising from the CW geometry is especially important to model in investigations of symptomatic CWs (*58*).

Increased transverse and time-averaged WSS at the CW may also contribute to symptomatic CWs. The combined effect of these flow patterns suggests that the highly variable WSS metrics at the CW may lead to endothelial dysfunction via the inflammatory pathway, which, together with the low recirculating flow in the CW pocket, may contribute toward the thrombogenesis of coagulative blood clots. Larger CW angles also enhance thrombotic risk, showing pronounced thrombus formation for webs >60° in angle, with increased flow disturbance and up to 10% increase of maximum web stress. CW locations may also affect symptomatic thrombosis, as internal carotid artery CWs have been shown to generate the most flow disturbance and thrombus aggregation risk across carotid locations, with the common carotid artery (CCA) CWs being the least symptomatic (*59*).

Composed primarily of fibroelastic tissue and SMCs (*60*), CWs are thought to contribute to stroke predominantly through biomechanical mechanisms. CWs promote thrombosis through flow stasis distal to the web, which facilitates coagulation. In this distal recirculation zone, ECs perceive the loss of laminar shear through mechanosensors such as integrins, which may trigger a phenotypic transition toward a proinflammatory state, contributing to a thrombogenic environment for pathological clot formation. It is also possible that the disturbed flow leads to local inflammatory responses, which alter EC function, leading to increased collagen deposition and elastic lamina breakdown that preludes intimal fibromuscular dysplasia. The change in the local microenvironment may exacerbate the proliferation of fibrous and smooth muscle tissue in the intima, leading to the development of the CW. This is especially interesting given the similar microenvironment generated by collagen buildup to that of the medial ECM layer, where vascular SMCs generally reside, raising a potential explanation for intimal fibromuscular dysplasia, a condition which remains elusive in its etiology (*61*). Platelets, fibrin, red blood cells, and other coagulative factors accumulate in the recirculation zone, forming a clot that eventually fragments and embolizes into distal cerebral vessels, leading to a stroke. This thromboembolic pathway aligns with infarctions found in the cortical and deeper vascular regions of patients with CW. Of course, that is just one possibility. Clots may also form upstream of the carotid bifurcation and get lodged at the CW lumen narrowing, although this is a lower risk, as 94.6% of patients with CW have been shown to have a normal lumen with no stenosis. Most symptomatic CWs have been shown to have noncritical <50% stenotic rates (*62*).

To properly capture such complex hemodynamics, in vitro models should replicate the flow regimes and vascular structures of the cerebral venous sinus and carotid webbed arteries to investigate the biomechanical factors that influence thromboembolism in these structures.

### Hemodynamic shortcomings of current CVS and CW in vitro models

Compared to in vivo and in silico models, in vitro models have the benefit of incorporating all three components of Virchow’s triad in a single model. Extensive control over biological and mechanical variables enables precise modeling of human and patient-specific vascular conditions. Over the past two decades, diverse fabrication techniques have been employed to develop vascular platforms, including extrusion-based 3D printing, soft lithography, and planar microfluidics, and polydimethylsiloxane (PDMS) casting of rigid microchannels (*63*). Each has contributed insights into vascular biology, coagulation dynamics, and drug testing. However, when applied to complex vascular structures such as CVST and CW, they fail to replicate the anatomical and hemodynamic environments characteristic of these conditions.

Existing in vitro systems remain limited in their ability to reproduce the complex hemodynamic environments that govern thrombosis in CVST and CW. Soft-lithographic and PDMS-based platforms typically use planar or cylindrical channels that neglect curvature and multibranch connectivity, leading to oversimplified laminar flow profiles. Extrusion-printed (*64*) constructs provide more geometric freedom but still lack the precision and surface resolution required to recreate patient-specific sinus geometries. Moreover, stiffness mismatches between PDMS and dural tissue distort hemodynamics, further reducing physiological relevance.

A recent advance by Zhao *et al.* (*54*, *55*) addressed several of these challenges through the creation of a “movable-typing” vein-on-chip, a stereolithography-based microfluidic model that reconstructed the sigmoid sinus geometry from a CVST patient’s MRV image. Their design successfully integrated all three components of Virchow’s triad: hemodynamics, endothelial function, and coagulability within an endothelialized, perfusable platform. The study demonstrated that fibrin-rich venous thrombi form in a shear-independent manner, distinct from arterial thrombosis, and that platelet adhesion occurred uniformly across the venous wall, independent of stenosis severity. This model, therefore, represented the first systematic attempt to correlate patient-specific geometry, endothelial activation, and blood coagulation in a controlled microfluidic environment.

However, the vein-on-chip also highlights the limitations of current in vitro CVST models. While it faithfully replicated a single sinus segment, the system relied on PDMS-based casting and planar assembly, constraining its ability to reproduce the full 3D branching and intersinus connectivity inherent to the cerebral venous network. Moreover, although large-scale fabrication was achieved, fine geometric fidelity was limited by the resolution of stereolithography and by postprocessing steps such as polishing and demolding. As Zhao *et al.* (*55*) acknowledged, achieving true anatomical fidelity, low-shear hemodynamics, and high-throughput scalability within a single construct remains technically challenging.

A central constraint is fabrication feasibility at physiologically relevant lumen scales. Enclosed microchannels below several hundred micrometers approach the practical resolution limits of soft lithography and stereolithographic printing, where light scattering, resin overcuring, and incomplete removal of uncured material compromise dimensional accuracy and patency. Multiplanar and branching architectures, such as the superior sagittal and transverse sinus network or intraluminal protrusions characteristic of a CW, further exacerbate these challenges due to difficulties in demolding, surface finishing, and maintaining structural integrity without collapse. As a result, many platforms adopt simplified or enlarged geometries that deviate from native hemodynamic conditions, particularly in low-shear venous environments, where small geometric perturbations critically influence thrombus initiation.

The absence of patient-specific anatomical fidelity remains the most critical shortcoming. CVST risk is influenced by interindividual variations in sinus geometry, including degree of tapering, curvature, and angulation, yet most models are based on idealized or symmetric designs. These simplifications obscure the relationship between vascular structure and disease susceptibility and hinder the development of personalized therapeutic strategies. This limitation is particularly important given that, although anticoagulation remains the standard treatment, clinical outcomes are highly variable, with some patients exhibiting delayed or incomplete recanalization or progression despite therapy, suggesting a critical role for local hemodynamics and geometry in treatment response. Recent studies recognize this gap, noting that achieving realistic, scalable, and perfusable reconstructions of human venous anatomy remains a primary technical barrier (*55*, *65*).

This issue is even more pressing with CWs. To our knowledge, all studies on the biomechanical effects have been conducted in silico, with no physical in vitro models reported. These simulations are based on patient-specific carotid artery images and typically use rigid wall simulations for more rapid computation. However, rigid wall simulations underestimate thrombus areas by 15.7 to 85.7% for CW structures (*66*), limiting their benefit for the accurate diagnosis of symptomatic CWs. Such computational studies support an advanced clinical management framework for CW, including a turbulence intensity–based scoring system (*67*), a CFD stroke risk assessment tool (*68*), and surgical recommendations. However, their clinical applicability is limited by the lack of biological validation. Although hemodynamic metrics may indicate key biomechanical mechanisms leading to thrombosis and stroke, the actual responses of blood components and vascular cells remain unclear.

### Technical challenges in replicating CVST and CW biological mechanisms

In addition to the anatomical and hemodynamic challenges for in vitro models, the biology of human vessels is difficult to reproduce. First, to faithfully reproduce the cellular structure of the vascular wall, vascular ECs must generate a confluent layer on the innermost surface, similar to the tunica intima. 3D channel networks can be difficult to perfuse uniformly due to nonuniform flow patterns in branched channels, where channel resistance can affect perfusion rates and volumes. As a result, this may lead to disorganized cellular distribution across the channels and different cellular morphologies if made to differentiate and mature under flow, such as the case for induced pluripotent stem cell–derived ECs (iPSC-ECs) (*69*). Second, various EC sources exist, each with different component protein expressions and biomechanical responses. Generic EC cell lines exist, such as human umbilical vein ECs (HUVECs) and iPSC-ECs (*70*). Therefore, the choice of ECs for CVST and CW models has to be carefully considered with a suitable cell handling regime developed. In addition, replicating the multilayer vascular wall structure with SMCs and collagen in the tunica media and an elastic tunica adventitia may be challenging. With regard to the CW specifically, the challenge lies in seeding ECs uniformly in the presence of an angled protruding flap, while keeping the flap itself cell-free in line with the lack of ECs observed in in vivo CWs (*61*).

After creating cellularized vascular channels, the in vitro vascular models will be used for blood perfusion experiments, where the hemodynamic parameters are reproduced using controlled flow regimes to model biomechanical responses of the seeded ECs or thromboembolic processes of the perfused blood (*71*, *72*). Although patient blood may provide the most physiologically relevant representation of disease-specific conditions for patient-specific modeling, obtaining it for research can be difficult, particularly when relying on clinically collected samples. The use of whole blood from healthy donors can be used to investigate biomechanical responses and thromboembolic progression in healthy patients, suitable for use in CW modeling, as patients generally lack other cardiovascular risk factors. However, even with healthy whole blood, donor variability may lead to unaccounted-for differences in thrombotic responses (*73*, *74*). Handling samples for whole blood thrombosis assays also has typical challenges of anticoagulant reversal, platelet function drift, temperature, and storage effects.

Although human and animal models may offer greater biological fidelity and overcome such challenges, the characteristics of CVST and CW render such models insufficient as well. Randomized, controlled clinical trials are difficult to conduct for CVST and CW due to small patient populations (*75*). Animal models, although a common alternative to clinical trials, are particularly unsuitable because CW are at present unverified in animals, and surgically recreating or altering native animal vessels to simulate the CW structure is both technically challenging and unethical. Tissue-engineered vessel implantation in animal models introduces confounding influence effects from vessel material and cell constituents. Moreover, the pathophysiological mechanisms observed in animal models do not always fully recapitulate human disease, limiting the translational relevance of experimental findings (*63*). To overcome these biological, anatomical, and ethical constraints, emerging light-based 3D bioprinting strategies offer a promising framework for engineering high-fidelity, patient-specific cerebrovascular models.

## ENGINEERING IN VITRO PATIENT SPECIFIC CVST AND CW MODELS WITH LIGHT-BASED 3D BIOPRINTING

The development of cerebrovascular models builds upon foundational microstereolithography techniques, which first demonstrated the ability to pattern complex internal architectures and biochemical factors within polymer scaffolds (*76*). Subsequent advancements in microscale continuous optical bioprinting demonstrated the ability to directly encapsulate multiple vascular cell types within complex 3D architectures, promoting the spontaneous formation of lumen-like structures (*77*). Broadly, light-based bioprinting modalities leverage spatially controlled photopolymerization to achieve high-fidelity spatial resolution, isotropic mechanical properties, and exceptionally smooth internal surfaces that minimize artifactual flow disturbances. These capabilities are particularly advantageous for vascular modeling, where subtle variations in lumen morphology markedly influence local hemodynamics, shear stress gradients, and thrombus formation. In CVST and CW, disease initiation is governed by complex interactions between vascular geometry, blood flow, and endothelial responses within tortuous and highly patient-specific anatomical structures. Experimental models, therefore, require fabrication approaches capable of accurately reproducing these geometries while maintaining optically transparent, perfusable luminal architectures suitable for endothelialization and real-time flow studies (*78*).

### Technical benchmarking of light-based modalities

DLP and VBP represent the two primary lithographic strategies for fabricating enclosed, perfusable cerebrovascular conduits with high geometrical fidelity. While both modalities leverage light-induced radical polymerization, their underlying spatial illumination profiles, cross-linking kinetics, and cell-biomaterial interaction dynamics differ fundamentally, introducing distinct trade-offs for modeling complex pathologies like CVST and CW. The selection of a specific light-based modality is dictated by a critical trade-off between spatial resolution, fabrication speed, and structural integrity. High-precision techniques such as two-photon polymerization (2PP) offer unparalleled submicrometer resolution, yet they are often constrained by small build volumes that preclude the fabrication of centimeter-scale patient-specific models (*79*). Similarly, laser-induced forward transfer provides excellent cell viability for high-viscosity bioinks but lacks the integrated volumetric or layer-wise projection capabilities required to efficiently generate fully enclosed, perfusable 3D geometries.

Traditional layer-based techniques like SLA are limited by extended fabrication times that exacerbate cell-settling within low-viscosity bioinks, ultimately leading to nonuniform cell distribution, while continuous liquid interface production (CLIP) subjects delicate constructs to substantial suction forces during the build process, risking the deformation of submillimeter features such as the intraluminal shelf in CW. In contrast, DLP uses a digital micromirror device to project entire layers simultaneously, substantially reducing fabrication time and shear-mediated damage. VBP further evolves this paradigm by employing tomographic projections to generate monolithic constructs in seconds, achieving a seamless luminal topography essential for modeling (*80*). Table 3 describes the comprehensive technical benchmarking of these light-based bioprinting modalities, evaluating their performance across critical engineering and biological parameters.

**Table 3.** Comparative analysis of light-based bioprinting modalities for cerebrovascular modeling.

Feature	2PP	SLA/CLIP	DLP	VBP
Fabrication	Nonlinear point-wise	Point-wise (SLA)/continuous (CLIP)	Layer-by-layer	Tomographic
Lateral resolution	Submicrometer (<1 μm)	High (∼25 μm)	High (∼25–50 μm)	Moderate (∼50–100 μm)
Fabrication speed	Low	Moderate/high	High	Ultrahigh (seconds)
Mechanical isotropy	High (point-fused)	Anisotropic (interfacial)	Marginal anisotropy	High (monolithic, seamless)
Internal surface finish	Nanoscale (<10 nm)	Variable (15–90 nm)	Moderate (∼25 nm)	Minimal (layer-less)
Material elastic modulus	Broad (0.1–140 kPa)	Limited range	Broad (tunable compliance)	Broad (tunable compliance)
Translational relevance	Scale-limited	Risk of structural failure	Optimal (CW shelf fidelity)	Optimal (CVST continuity)
Ref.	(*79*)	(*83*)	(*77*)	(*80*)

### Principles and workflow of DLP and VBP

Having established the relative strengths of each modality, the underlying fabrication workflows and engineering principles of DLP and VBP are discussed below.

#### DLP bioprinting mechanics

DLP bioprinting is a light-based additive manufacturing approach in which each layer of a 3D construct is formed through spatially patterned photopolymerization of a photosensitive bioink. Rather than depositing material mechanically through a nozzle, DLP projects a two-dimensional light pattern onto the precursor solution, typically using a digital micromirror device or related projection system, to selectively cross-link the desired geometry layer by layer. This enables rapid fabrication with high lateral resolution, smooth luminal surfaces, and minimal shear-mediated damage to encapsulated cells, making it particularly attractive for engineering vascular constructs in which local geometry strongly governs hemodynamics (*54*, *55*, *65*, *81*, *82*). While traditional DLP involves discrete steps, the transition toward continuous and dynamic printing processes (*83*) has substantially enhanced the structural integrity of these constructs. By eliminating the physical boundaries between layers, these advancements enable the fabrication of high-fidelity, leak-free vascular architectures that are essential for long-term perfusion studies.

For cerebrovascular modeling, these capabilities are especially important. Conditions such as CVST and CW arise in vascular regions where subtle variations in curvature, tapering, junctional architecture, or intraluminal protrusions can substantially alter shear stress distribution, flow separation, and thrombotic risk. DLP is well-suited to this scale of modeling because it can reproduce enclosed and tortuous channel networks with micrometer-level precision while maintaining structural continuity across the lumen. DLP also allows fine control over each projected slice, which can be advantageous when tuning local geometry or wall thickness in patient-specific constructs.

The DLP workflow begins with material selection and formulation. Photocrosslinkable hydrogels such as gelatin methacryloyl (GelMA), poly(ethylene glycol) diacrylate (PEGDA), and hybrid polymer systems are selected based on optical transparency, cross-linking kinetics, water solubility, viscosity, and mechanical tunability, as well as their capacity to support endothelial culture where endothelialization is desired (*84*). Naturally derived matrices such as GelMA are particularly valuable in this context because they contain cell adhesive motifs that facilitate endothelial attachment and monolayer formation, whereas more bioinert systems such as PEGDA often require blending or functionalization to support vascular lining (*85*). For cerebral venous sinus modeling, bioink formulations are typically tuned to achieve elastic moduli in the low kilopascal range, reflecting the compliant mechanical environment of dural venous tissue while preserving sufficient structural stability for perfusion (*12*).

Once the bioink has been formulated, printing parameters must be carefully optimized to balance fabrication speed with structural accuracy. Critical variables include light wavelength, photoinitiator concentration, and oxygen inhibition effects, all of which dictate curing kinetics and interlayer fusion. However, in cell-laden bioinks, shape fidelity can be compromised by light scattering, where elevated cell concentrations act as refractive centers that disperse the projected light pattern and blur the intended geometry. While standard cell-laden formulations remain highly printable under typical conditions without intervention, ultra-high cell density constructs increasingly require advanced refractive index-matching strategies or optimized resin formulations to preserve fidelity in complex, large-scale vascularized constructs (*86*). Such refinements are essential for maintaining the integrity of small luminal channels and rapid caliber changes, where light attenuation must be strictly confined to ensure the printed architecture accurately reflects the patient-specific digital design.

In patient-specific applications, vascular geometries reconstructed from MRV, computed tomography, or machine learning-assisted segmentation pipelines are converted into a series of 2D masks that guide sequential light projection during printing (*87*). This enables direct translation of imaging-derived anatomy into experimentally tractable constructs while retaining the local geometric features that drive disease-relevant hemodynamics. After printing, constructs are typically washed to remove uncross-linked precursor material, endothelialized, and integrated with perfusion systems for controlled flow studies. DLP bioprinting combines rapid fabrication, high spatial precision, and reliable control over enclosed vascular geometry, making it particularly well suited to cerebrovascular modeling.

#### VBP tomographic kinetics

VBP represents a fundamentally different approach to light-based fabrication, in which 3D structures are generated within a photosensitive hydrogel volume in a single integrated step (*17*). Unlike DLP, where polymerization occurs almost instantly at a single focal plane, VBP relies on the spatial accumulation of a cumulative optical dose (*80*). The cross-linking reaction is strictly governed by a nonlinear threshold mechanism; the local concentrations of generated free radicals only trigger a localized gelation network in spatial coordinates where the cumulative light energy exceeds a critical gelation threshold (*88*). In regions where the accumulated energy remains below the gelation threshold, the bioink stays entirely liquid, acting as a natural, buoyant support matrix that eliminates the need for sacrificial temporary scaffolds. This allows the direct fabrication of continuous, tortuous multibranched channel configurations within seconds.

The volumetric workflow begins with similar material considerations but places emphasis on optical homogeneity and transparency as well as controlled photopolymerization kinetics. Bioinks must exhibit minimal light scattering and predictable dose response behavior to ensure spatial accuracy during volumetric exposure (*80*). GelMA, PEGDA, and hybrid formulations are commonly adapted for volumetric printing by fine-tuning polymer concentration, photoinitiator efficiency, and viscosity (*89*). Compared to nozzle-based deposition methods, VBP is not constrained by filament formation during printing, which, in principle, broadens the range of usable viscosities and allows construct formation without the need to maintain shape during extrusion. This is advantageous for soft vascular bioinks that would otherwise be difficult to process while preserving lumen continuity and geometric fidelity.

Patient-specific anatomical models are first computationally decomposed into a series of angular light projections that encode the complete 3D geometry. These projections are then delivered into a rotating bioink volume using spatially controlled light fields. As the projections accumulate, photopolymerization occurs selectively within defined regions of the hydrogel precursor, resulting in the formation of the desired structure throughout the volume simultaneously. This strategy enables the fabrication of highly complex geometries, including tortuous and enclosed vascular networks, within seconds to minutes (*80*). Such integrated volumetric curing is valuable for reproducing continuous hollow channels and branching architectures, where small geometric deviations can substantially affect hemodynamic behavior.

Material selection plays a critical role in the success of volumetric fabrication. Beyond the standard requirements for cytocompatibility, VBP is sensitive to the optical homogeneity of the precursor bioink. A fundamental technical constraint remains the light scattering caused by volumetric illumination, particularly in cell-laden systems (*90*). In this modality, encapsulated cells act as refractive centers that disperse tomographic light projections as they traverse the rotating reservoir, which compromises the spatial precision of the cumulative energy dose. This dispersion is particularly detrimental to the fidelity of delicate internal features, such as the branching architectures or the complex luminal protrusions characteristic of CVST. To preserve the integrity of these patient-specific geometries, current research prioritizes the development of bioinks with matched refractive indices, which harmonize the optical properties of the cellular phase and the hydrogel matrix to minimize light dispersion during the integrated curing process (*86*).

A key advantage of VBP is the ability to generate smooth, fully enclosed structures without the need for sacrificial support or sequential layer deposition. Because the construct forms within the liquid precursor volume, delicate internal features such as hollow luminal channels and branched vascular geometries can be produced directly with high structural continuity. This capability is particularly valuable for vascular modeling applications, where subtle geometric features and smooth internal surfaces strongly influence hemodynamic behavior and endothelial responses.

This rapid, monolithic curing mechanism provides profound biological advantages for cell encapsulation. Because the total processing time for a centimeter-scale construct is condensed into a brief window (20 to 60 s), VBP bypasses the cellular settling and nonuniform density gradients that plague slow, layer-by-layer printing (*91*). Cells remain perfectly suspended in their native 3D spatial coordinates during the brief curing phase, yielding unmatched postprint cell viability metrics. Furthermore, because VBP does not involve scraping, peeling, or window detachment forces, encapsulated cells are protected from shear-mediated membrane damage during the build, preserving their intrinsic mechanosensitive pathways before functional perfusion testing begins.

Despite these advantages, volumetric constructs typically require careful postprocessing following fabrication. After photopolymerization, uncross-linked precursor material remains surrounding the printed structure and within internal luminal spaces. Removal of this residual hydrogel is therefore necessary to reveal the final geometry and establish functional perfusable channels. Postprocessing is commonly achieved through gentle washing, secondary curing to ensure structural fidelity, controlled perfusion, or solvent exchange to extract uncured material while preserving the integrity of the printed matrix (*92*). Appropriate optimization of these steps is critical, as aggressive washing or prolonged exposure to extraction solvents can compromise delicate hydrogel architecture or alter the mechanical properties of the construct (*93*).

Through the combination of rapid fabrication, high geometric fidelity, and the ability to generate enclosed vascular architectures, VBP has emerged as a powerful platform for engineering complex 3D tissue constructs. In the context of cerebrovascular disease modeling, these capabilities provide a compelling route for recreating patient-specific venous sinus networks and carotid bifurcation geometries in vitro, enabling experimental investigation of hemodynamic and mechanobiological processes that underlie thrombosis.

Figure 3 illustrates the DLP and VBP workflow used to generate patient-specific vascular models. Segmented medical imaging data are converted into projection masks that guide layer-wise photopolymerization of photocrosslinkable bioinks, enabling fabrication of enclosed perfusable venous geometries with high spatial resolution. Following printing, constructs are washed to remove uncross-linked material and connected to perfusion systems for controlled flow studies. ECs can be seeded after fabrication or incorporated within the hydrogel matrix, allowing investigation of endothelial mechanotransduction, thrombus initiation, and clot propagation under physiologically relevant venous hemodynamics. Subsequent structural and functional characterization ensures that printed constructs reproduce key anatomical and biomechanical features of the target vasculature. These validated platforms can then be applied as experimental models to study disease mechanisms and evaluate patient-specific hemodynamic risk factors, providing a translational bridge between imaging data, laboratory investigation, and clinical decision support. Figure 3 (C to E) illustrates the volumetric fabrication principle of CAL, which addresses several core limitations of traditional layer-by-layer 3D printing. As shown in the schematic of the CAL mechanism (Fig. 3C), the process uses a rotating volume of photosensitive resin that is subjected to multidirectional, dynamically evolving light patterns. By using a DLP as the primary illumination source (Fig. 3D), a calculated 3D optical dose is delivered simultaneously to the entire target geometry. This volumetric approach offers a distinct advantage in terms of fabrication speed; as demonstrated in the time-lapse progression (Fig. 3E), complex centimeter-scale objects can be solidified in under 60 s. Because the object is formed concurrently within the resin volume, this method overcomes the need for physical support structures, as the surrounding uncured resin provides natural buoyancy for the developing part. The technique is also highly adaptable to varied material compositions, including the incorporation of opaque dyes, and remains scalable for larger, high-fidelity structures without compromising the integrity of the 3D reconstruction.

**Fig. 3.**
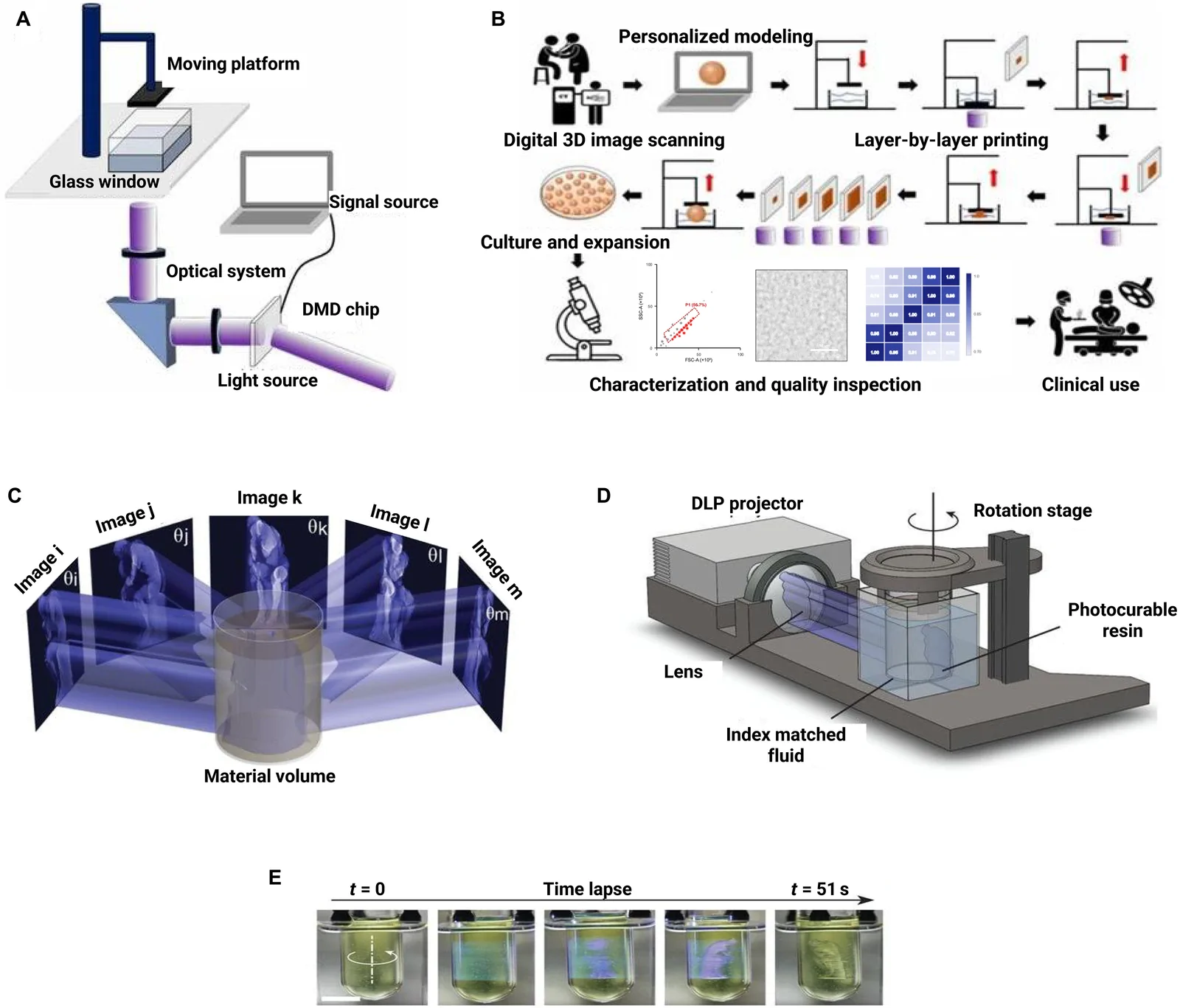
DLP and VBP bioprinting workflow for fabrication of patient-specific cerebrovascular models. (**A**) Schematic of a DLP-based three-dimensional bioprinter. (**B**) Translational workflow for patient-specific model development. (**C**) Schematic of computed axial lithography (CAL) VBP multidirectional patterned illumination into photosensitive resin. (**D**) Diagram of the experimental setup with a digital light processor (DLP) projector. (**E**) Time-lapse progression showing the rapid solidification within the resin volume in under 60 seconds. [(A) and (B) adapted with permission from (*119*), Copyright IOP Publishing Ltd.; (C), (D), and (E) adapted with permission from (*92*), Copyright AAAS].

### Integrating advanced biomanufacturing with dynamic materials for biomimetic cerebrovascular models

The success of light-based fabrication does not rest solely on geometry but also on the materials chosen to construct these models. GelMA has become the most widely used photopolymer due to its tunable stiffness, degradability, and cell-adhesive motifs. It is generated by methacrylation of gelatin, which introduces photocrosslinkable groups that enable light-induced polymer network formation during fabrication. By adjusting polymer concentration and degree of substitution, elastic moduli ranging from 1 to 20 kPa can be achieved, spanning the range that supports endothelial quiescence and shear responsiveness (*94*). Blends with synthetic polymers such as PEGDA or thiol-ene systems further allow wall regions to be stiffened while retaining softer luminal surfaces conducive to endothelial lining. Photoabsorbers such as tartrazine can attenuate light depending on the absorbance and light wavelength, preserving lumen fidelity at junctions where caliber changes rapidly. For biological fidelity, these constructs can be endothelialized with ECs or with endothelium derived from iPSCs. Under physiologic venous shear stresses of 1 to 6 dyn cm^−2^, these cells align with flow, reorganize junctional proteins, and regulate nitric oxide and vWF secretion, reproducing venous endothelial behavior. Coculture with fibroblasts or pericyte-like cells in the surrounding matrix enhances barrier integrity and more closely mimics the dural sinus wall, which combines a rigid outer structure with a biologically active lining. Emerging dynamic materials, including phototunable hydrogels and enzymatically degradable cross-linkers, add further flexibility by allowing patterned stiffness and controlled remodeling during perfusion (*95*). Table 4 summarizes representative photocrosslinkable biomaterials used in light-based cerebrovascular bioprinting, with their cross-linking mechanisms, stiffness ranges, and light wavelengths.

**Table 4.** Photocrosslinkable biomaterials for light-based bioprinting.

Material system	Typical macromer conc. (w/v)	Wavelength (nm)	Photoinitiator conc. (w/v)	Photoabsorber conc. (w/v)	Young’s modulus (*E*, kPa)	Ref.
GelMA	5–15%	365–405	LAP: 0.05–0.25%, Irgacure 2959: 0.1%	Tartrazine or quinoline yellow: 0.01–0.05%	0.5–30	(*77*, *79*)
PEGDA	10–20%	365–405	LAP: 0.1–0.5%	Tartrazine: 0.02–0.1%	20–150+	(*83*, *90*)
HAMA	1–3%	365–405	LAP: 0.05–0.1%	Tartrazine: 0.01%	0.1–5	(*91*, *132*)
GelMA and HAMA	5–10%	365–405	LAP: 0.1%	Tartrazine: 0.015%	1–15	(*95*, *105*)
GelMA and PEGDA	5–10%	365–405	LAP: 0.1–0.2% or Ru/SPS: 1/2 mM	Quinoline yellow: 0.02%	2–80	(*89*, *105*)

Photoinitiators are central to light-based bioprinting as they govern the initiation and spatial control of polymer network formation during photo-induced cross-linking. In bioprinting, photoinitiator selection must balance efficient radical generation with cytocompatibility, optical penetration depth, and uniform cross-linking within enclosed and patient-specific geometries. Lithium phenyl 2,4,6 trimethylbenzoylphosphinate (LAP) has emerged as one of the most widely used photoinitiators due to its high water solubility, relatively low cytotoxicity, and strong absorption in the near ultraviolet to visible range compared with earlier initiators such as Irgacure 2959 (*96*). LAP exhibits substantially greater molar absorptivity near visible wavelengths, with reported extinction coefficients on the order of ∼30 M^−1^ cm^−1^ at 405 nm, whereas Irgacure 2959 (*96*) displays negligible absorbance (<5 M^−1^ cm^−1^) at this wavelength and is primarily activated under ultraviolet irradiation around 365 nm. This difference enables efficient radical generation using 405-nm light sources common to DLP and VBP systems, driving rapid polymerization while avoiding cell-damaging ultraviolet wavelengths.

Adjusting the operational concentration of these photoinitiators serves as a primary lever for tuning the matrix’s final Young’s modulus. While the macromer concentration establishes the baseline structural potential, the photoinitiator concentration acts as the active control switch. For instance, raising the LAP concentration from 0.05 to 0.25% (w/v) increases the number of free radicals generated per unit volume under light exposure. This higher radical density creates more covalent bonds across the polymer chains, increasing cross-linking density (*97*). This mechanism allows the use of the same base material formulation to produce either highly compliant, low-modulus geometries mimicking thin-walled venous channels, or the much stiffer, higher-modulus matrices required for high-pressure arterial walls.

While photoinitiators control the kinetics of cross-linking, photoabsorbers play a complementary role by shaping the optical field during fabrication. Photoabsorbers are commonly used in DLP-based bioprinting, where layerwise light projection can otherwise cause excessive penetration, unintended curing, and loss of vertical resolution (*98*). By introducing defined concentrations of biocompatible photoabsorbers such as tartrazine or quinoline yellow, the dye acts as a competitive light sink that restricts polymerization strictly to the targeted focal plane. This precise balance between photoinitiator radical generation and photoabsorber light attenuation prevents vertical overcuring and subsequent light leakage. Controlling this photokinetic balance is particularly important for maintaining internal lumen patency and reproducing the complex curvatures, gradual diameter transitions, and junctional regions characteristic of cerebrovascular anatomy (*99*).

Ultimately, the architectural and operational versatility of light-based bioprinting extends far beyond individual vascular anomalies, serving as an expansive, high-utility platform for diverse organ-on-chip disease modeling and advanced biomaterials exploration. They provide highly adaptable fabrication where layer-by-layer and tomographic projections can be matched to the distinct structural scale and physiological demands of varied organ systems. This technological flexibility enables the high-fidelity reconstruction of multiple pathological environments, ranging from the delicate, submillimeter intraluminal features of localized stroke shelves to the highly tortuous, macroscale dural networks of venous sinuses, and even extending to multiscale capillary networks required for modeling tumor angiogenesis or blood-brain barrier permeability (*90*). By dynamically modifying light intensity profiles or exposure masks mid-cycle, it is possible to establish precise cross-linking gradients within a single integrated platform, seamlessly blending structurally supportive hydrogel bulk regions with highly compliant, specialized matrices that replicate local mechanical niches. This capacity enables the exploration of multimaterial formulations, such as embedding cell-adhesive GelMA or glycosaminoglycan-rich hyaluronic acid arrays within elastomeric synthetic networks, to systematically investigate how local tissue stiffness influences downstream cellular pathology (*77*).

### Functional characterization and flow-driven application of bioprinted vessels

This fundamental intersection between fabrication versatility and localized disease configurations dictates that bioprinted structures must be functionalized via dynamic fluidic environments to achieve true physiological translation. Transforming biomaterial frameworks into highly responsive, predictive in vitro platforms requires the integration of continuous fluid perfusion, regulated hemodynamics, and multicellular wall architectures that can replicate native in vivo vascular niches. Building upon the light-based fabrication strategies presented in Fig. 3, Fig. 4 highlights representative translational applications that demonstrate how these technologies can be integrated with perfusion systems and biomimetic vascular environments to generate physiologically relevant neurovascular disease models.

**Fig. 4.**
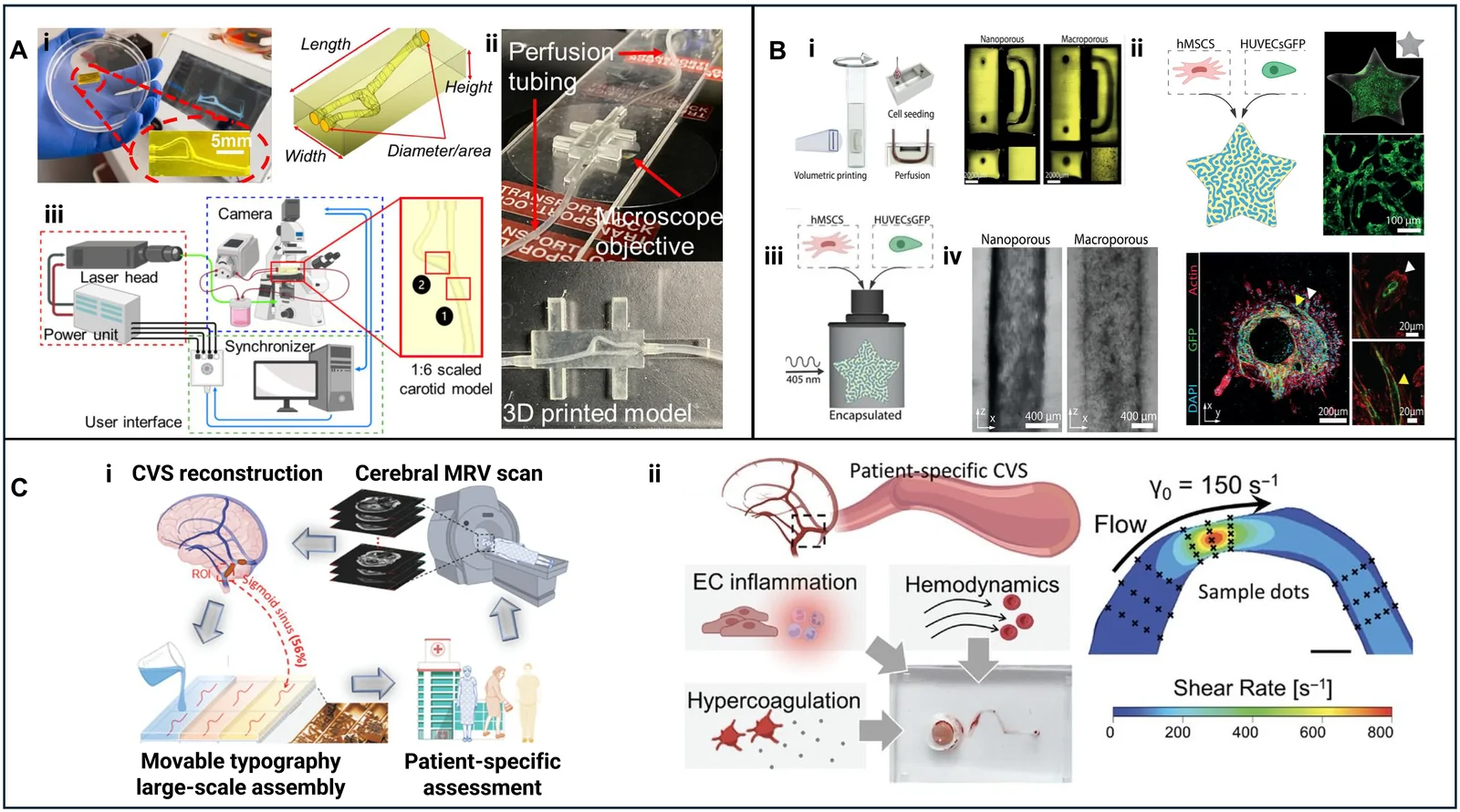
Light-based biofabrication applications for vascular disease modeling. (**A**) DLP photopolymerization framework translating patient-derived carotid bifurcation geometries into continuous hydrogel networks. J. A. Catano, L. J. Y. Ong, M. Yuan, Y. Qu, J. Benitez, P. K. Yarlagadda, Y. Toh, Z. Li, A Patient-specific 3D printed carotid artery model integrating vascular structure, flow, and endothelium responses. Advanced Healthcare Materials. (2026). 15, e02478. © 2025 The Author(s). Advanced Healthcare Materials published by Wiley-VCH GmbH. (**B**) VBP paradigm creating photocrosslinkable hydrogel constructs using various endothelialization strategies to initiate endothelial sprouting and perivascular maturation. Adapted under a Creative Commons CC-BY 4.0 License from (*120*). (**C**) Modular template casting and hemodynamic mapping for patient-specific “vein-on-chip” models and hemodynamic evaluation using CFD. Adapted under a Creative Commons CC-BY 4.0 License from (*55*).

Across the broader biomedical landscape, the application of microfluidic configurations and biomimetic vascular networks has fundamentally shifted the paradigms of disease modeling, therapeutic screening, and surgical intervention. In safety and efficacy pharmacology, these functionalized vessels serve as automated screening arrays for evaluating real-time drug toxicities and anti-angiogenic tumor therapeutics. In regenerative medicine, perfusable vascular networks serve as living supply lines embedded within thick, dense cellular constructs, overcoming mass-diffusion limitations to ensure nutrient delivery and metabolic waste clearance, thereby supporting long-term parenchymal survival (*100*). Recent advances in vascular biofabrication have further demonstrated the fabrication of mechanically robust, hierarchical vascularized constructs capable of sustaining physiological perfusion and direct vascular anastomosis following implantation, highlighting continued progress toward clinically translatable engineered tissues and long-term functional integration (*101*). Together, these advances illustrate that vascular biofabrication is evolving beyond the fabrication of anatomically accurate constructs toward the development of durable, functional vascular systems suitable for translational applications. Concurrently, light-based volumetric and lithographic bioprinting strategies have enabled the rapid generation of seamless, multibranched vascular conduits using advanced photocrosslinkable hydrogel systems, including photo click chemistries, hydrogels that preserve mechanical compliance and luminal patency under continuous dynamic shear, supporting long-term endothelial maturation and barrier integrity without structural failure (*102*).

Most critically, these specialized fluidic configurations offer animal-free methodology to simulate the specific, shear-mediated molecular mechanisms driving human vascular pathologies, serving as a cornerstone for patient-specific diagnostics and precision medicine. In pathobiological disease modeling, microfluidic channels allow isolation of the individual or coupled components of Virchow’s triad by systematically varying flow rates, introducing circulating inflammatory biomarkers, or altering the EC matrix composition (*58*). This capacity to modulate fluid forces is particularly vital when modeling disorders governed by altered hemodynamics, where localized stasis, boundary layer separations, or extreme shear gradients act as direct physical cues that trigger procoagulant cascades, cellular remodeling, or focal intimal hyperplasia.

Figure 4 illustrates the vascular modeling applications of various light-based modalities. In particular, Fig. 4A demonstrates DLP printed carotid bifurcation models that allow for real-time, noninvasive optical monitoring and empirical velocity vector mapping of complex flow physics in carotid artery disease. Figure 4B shows how tomographic projections of the vascular architectures can be used to construct monolithic photocrosslinkable hydrogel conduits with multiscale perfusable channels and interconnected microporosity. It demonstrates the progression from initial channel architecture and cellularization strategies by comparing postprint surface seeding against in situ tomographic encapsulation to dynamic bioreactor perfusion that drives human mesenchymal stem cell-led lumenized endothelial sprouting and perivascular maturation throughout the macroporous matrix. Figure 4 illustrates the translation of patient MRV scans into reconstructed venous geometries to construct “vein-on-chip” models of the sigmoid sinus junction with CFD to track localized shear rate gradients and evaluate how the synergistic forces of endothelial inflammation, localized stasis, and hypercoagulability trigger CVST. Together, these techniques show promise in developing perfusable, in vivo relevant vascular models for studying various cerebrovascular conditions.

### Technical challenges in bioprinted cerebrovascular models

Despite rapid advances in light-based biofabrication technologies, several technical and biological barriers continue to limit the development of physiologically faithful cerebrovascular models. A central challenge lies in simultaneously achieving high printing resolution while maintaining scalability and fabrication throughput. Many advanced optical bioprinting systems can generate microscale and submicroscale structural features necessary to reproduce fine vascular geometries. However, preserving such resolution often conflicts with the need for rapid volumetric fabrication and high-throughput production of large three-dimensional constructs. Increasing printing speed or construct size can reduce spatial precision, while strategies that maximize resolution frequently require slower processing or restricted build volumes. Balancing resolution, fabrication speed, and construct scale, therefore, remains a key engineering constraint in vascular bioprinting (*103*).

Material design presents an additional challenge. Cerebrovascular constructs frequently require multimaterial architectures in which different regions of the model exhibit distinct mechanical or biological properties. For example, luminal surfaces must support endothelial adhesion and shear responsiveness, whereas surrounding matrices may require higher stiffness to maintain structural stability. Streamlined multimaterial processing remains difficult in many light-based printing platforms because sequential material exchange can disrupt optical alignment or introduce severe interfacial delamination artifacts. Because adjacent hydrogel zones often possess differing cross-linking kinetics and differential water-driven swelling ratios, continuous perfusion workloads risk shearing the construct apart at the material seams. Overcoming this hurdle requires the development of photo-responsive resins capable of maintaining covalent cross-linking continuity across multimaterial interfaces to effectively replicate distinct cellular wall boundaries without structural failure (*104*).

Fabrication limitations are also closely linked to physical postprocessing artifacts that compromise patient-specific geometric fidelity. Following photopolymerization, uncross-linked precursor material must be thoroughly flushed from the internal luminal spaces. In low-modulus, highly compliant hydrogel matrices, this extraction phase frequently introduces localized matrix shrinkage and structural warping due to abrupt capillary pressure changes and osmotic gradients. These subtle physical distortions alter internal conduit cross sections, inducing unvalidated hydrostatic pressure variations during flow trials that can ultimately mask real-world cellular mechanobiology (*105*).

Another fundamental physical limitation shared by both techniques when printing cell-laden systems is the optical dispersion of highly refractive cells in aqueous hydrogel precursors. To overcome this optical barrier and maintain the geometric fidelity required for patient-specific cerebrovascular models, advanced biofabrication frameworks implement refractive index matching (*86*). By dissolving biocompatible, nonionic iodinated contrast agents into the bioink formulation, the refractive index of the continuous liquid phase can be precisely elevated to perfectly match the cellular phase, allowing the projected light fields to pass through cell-dense hydrogel volumes with minimal scatter.

These challenges highlight the need for integrated advances in fabrication physics, material chemistry, and vascular cell biology. Future progress in light-based biofabrication will likely depend on the development of next-generation bioinks with programmable mechanics, improved multimaterial printing workflows, and refined perfusion strategies that enable stable endothelialization and reproducible hemodynamic conditions.

## AN APPROACH TO IN VITRO MODELING FOR CVST AND CW

### A framework for CVST and CW modeling

On the basis of our understanding of CVST and CW, as well as the limitations and capabilities of various biofabrication techniques, we can develop a framework for modeling these conditions. This section outlines a systematic framework for modeling CVST and CW in vitro, defining key mechanistic targets, cell sources, fabrication parameters, and experimental readouts. The proposed framework is summarized in Table 5.

**Table 5.** Recommended approaches to modeling CVST and CW. HCtAECs, human carotid artery endothelial cells; HBMECs, human brain microvascular endothelial cells; HUVECs, human umbilical vein endothelial cells; SMCs, smooth muscle cells; AAW, African American women; CaW, Caucasian women; AW, African women; MK, midkine; α-SMA, α-smooth muscle actin; CCA, common carotid artery; WSR, wall shear rates.

Technical and biomechanical features	CVST	CW (development)	CW (thrombosis)	Ref.
Mechanism	Venous stasis, endothelial activation, and hypercoagulability (Virchow’s triad) leading to venous thrombosis	Complex hemodynamic flow and hormonal fluctuations potentially leading to endothelial activation, subendothelial remodeling, and abnormal SMC proliferation	Recirculating flow in the web pocket and highly variable local shear leading to thromboembolic developments	(*10*, *12*)
Cell coating choice	HUVECs or HBMECs	HCtAECs & vascular SMCs	HCtAECs	(*55*, *82*)
Fabrication techniques	**VBP:** 100 μm resolution	**DLP (bioprinting and VoC):** 10 μm resolution		(*80*, *82*)
	**DLP:** 25–50 μm resolution			
Key printing parameters	**VBP (bioprinting):** Wavelength: 405 nm; light dose: 200–230 mJ/cm²; average light intensity: 6 mW/cm²; print time: 20–40 s; steps per rotation: 1250; peak-to-average ratio: 7.0:1; high-viscosity resin with a low concentration photoinitiator	**DLP (VoC):** Wavelength: 405 nm; average light intensity: 25–100 mW/cm²; print time: 1–2 hours; layer thickness: 5–20 μm; exposure time: ∼1 s; low-viscosity resin		(*78*, *80*, *103*)
	**DLP (bioprinting):** Wavelength: 405 nm; average light intensity: 24 mw/cm²; print time: 25–35 min; layer thickness: 50–100 μm; exposure time: 8 s; low-viscosity resin with a higher concentration of photoinitiator			
Material systems and properties	Viscoelastic, soft hydrogel matrix with compressive modulus (stiffness): 2–10 kPa (matches compliant dural sinus); volumetric swelling ratio: 5–10%; stability: >14 days under continuous low-pressure perfusion (<1 Pa)	PDMS substrate at 25:1 (PDMS: curing agent) w/w ratio. Young’s modulus (stiffness): ∼230 kPa (matches young carotid arteries)		(*104*, *105*)
Protein and cell densities and ratios	**Monolayer seeding:** 1–10 million/ml (HUVECs/HBMECs) optimized to achieve a confluent, quiescent venous endothelial lumen	**Cocultured monolayer seeding:** 1–10 million/ml (vascular SMCs), followed by 50k/μl (HCtAECs)	**Monolayer seeding:** 1–10 million/ml (HCtAECs) optimized to achieve a confluent arterial endothelial lumen	(*55*, *133*)
		**Protein coating:** Collagen before and after seeding SMCs	**Protein coating:** Collagen before seeding HCtAECs	
Experimental variables	Venous sinus geometries (superior sagittal sinus, transverse sinus)	Estrogen hormone levels (premenopausal levels: 15–400 pg/ml, postmenopausal levels: 10–30 pg/ml)	CW geometries (angle, length)	(*12*, *44*, *48*, *55*, *134*)
	Blood flow velocities (0.05–0.15 m s⁻¹) and venous wall shear rates (1–50 s⁻¹ physiological)	Estrogen fluctuation patterns (slow decline, flat, rise/slow decline, rise/steep decline & amplitudes (AAW menstrual cycle levels: 50–225 pg/ml, CaW menstrual cycle levels: 50–190 pg/ml, AW menopause transition range: 160–35 pg/ml, CaW: 140–28 pg/ml)	Stenosis levels (noncritical: <50%, middle severity: 50–70%, critical: >70%)	
	Hormonal profiles: Premenopausal levels, ethinyl estradiol (EE) exposure (low-dose: 10-30μg; high-risk: ≥ 50μg) Endogenous Estradiol (E2) pregnancy peaks (5000-20,000 pg/ml), IVF spikes (>3000 pg/ml), or acute postpartum crashes (<30 pg/ml).		Blood flow rates & wall shear rates (mean CCA WSR: 260–500 s⁻¹, low WSR: <100–200 s⁻¹, high WSR: 8000 s⁻¹)	
	Postmenopausal levels, baseline castrate E2 limits (<10-20 pg/ml) paired with oral HRT exposure spikes or solid tumor malignancy hypercoagulable factors			
Observational variables	Abnormal cell growth and cell migration (venous alignment shifts, actin cytoskeleton remodeling under hydrostatic pressure)	Abnormal cell growth and cell migration	Clot formation and development (time to clot, size of clot)	(*49*, *53*, *68*, *100*, *133*, *135*–*137*)
	Expression levels of vascular wall biomarkers (localized tissue surface expression of TF and vWF vs. fluid perfusate shedding of IL-6 and TNF-α	Expression levels of vascular wall biomarkers (e.g., TF, MK)	Clot compositions (platelets, fibrin, RBCs)	
	Clot formation and development (Real-time total luminal occlusion velocity and time to clot)	Blood levels of thrombosis biomarkers (e.g., vWF, inflammatory cytokines IL-6, TNF-α)	Embolism frequency and size	
	Clot compositions (fibrin-rich red thrombus dense with captured RBCs)			
	Trans-endothelial barrier leakage: Continuous permeability tracking using 40–70 kDa FITC-dextran curves to evaluate loss of barrier integrity			
Validation criteria	Channel flow CFD validation: Venous flow and shear stress 0.05–0.15 ms^−1^ (physiological). Venous sinus shear rates 8 s^−1^ (downstream), 500 s^−1^ (upstream).	EC health and distribution: Clear, confluent morphology on imaging using CD31 (membrane), VE-cadherin (cell junctions), F-actin phalloidin (actin cytoskeleton), and Hoechst (nuclei) antibody labeling.	Channel flow CFD validation: Arterial shear rates 260–500 s^−1^ (physiological), <100–200 s^−1^ (low), 8000 s^−1^ (high). Flow recirculation pockets observed post-CW flap.	(*55*, *78*, *106*, *107*, *112*, *138*–*140*)
	EC health: Clear, confluent morphology on imaging using CD31 (membrane) and Hoechst (nuclei) antibody labeling.	SMC health and distribution: Clear, confluent morphology on imaging using α-SMA antibody labeling.	EC health: Clear, confluent morphology on imaging using CD31 (membrane), VE-cadherin (cell junctions), F-actin phalloidin (actin cytoskeleton), and Hoechst (nuclei) antibody labeling.	
	Junctional Integrity: Continuous, unbroken cell-to-cell borders confirmed via high-resolution VE-cadherin imaging along complex dural curvatures.	Activated EC biomarker detection: Biomarker detection on imaging using anti-TF and anti-MK antibodies.	EC inflammation: Biomarker detection on imaging using ICAM-1, VCAM-1, and TF antibodies.	
	EC inflammation: Biomarker detection on imaging using ICAM-1, VCAM-1, and TF antibodies.	Vascular wall thrombogenicity: Comparison of biomarker levels in blood/plasma measured before and after perfusion (24–48 hour incubation) using anti-vWF, TNF-α, and IL-6 ELISA kits (*P* > 0.05).	EC shear responsiveness: Biomarker detection on imaging using KLF2 and eNOS antibodies.	
	EC shear responsiveness: Biomarker detection on imaging using KLF2 and eNOS antibodies.		Platelet activation & aggregation: Biomarker detection on imaging using P-selectin and PAC-1 (GPIIb/IIIa) antibody.	
			Fibrin deposition: Biomarker detection on imaging using 59D8 anti-fibrin antibody.	
			RBCs: Biomarker detection on imaging using CD235a.	

The cerebral venous sinuses are rigid (*12*), noncompliant, dural channels whose multiaxial, curved geometries govern low-pressure, low-velocity venous flow (0.05 to 0.15 m/s), creating tapering networks like the torcular Herophili that generate localized stagnation pockets and low WSS prone to stasis-driven thrombosis (*52*). Capturing these large, continuous fluid domains without layer-by-layer delamination lines is essential to isolate the mechanisms of Virchow’s triad. For macroscale venous architecture, VBP serves as the primary fabrication modality to provide a seamless internal fluid path, while DLP can be deployed for highly localized venous features. To replicate dural wall compliance, the hydrogel is tuned to a compliant compressive modulus of 2 to 10 kPa with a restricted swelling ratio of 5 to 10% (*105*). This material stability prevents channel deformation over a 14-day assay runtime, ensuring that observed clot propagation is driven purely by patient-specific anatomy. To model the localized endothelial activation that precedes mural propagation, the internal lumen is coated with HUVECs or HBMECs at a standardized seeding density (1 × 10^6^ to 3 × 10^6^ cells/ml) to establish a quiescent monolayer (*55*).

Following endothelialization, the biological relevance of the printed sinus channels is verified through a multitiered functional characterization protocol designed to track barrier integrity and maturation along complex geometries. First, endothelial barrier integrity and permeability are quantified via continuous perfusate-based dextran leakage curves using 40 to 70 kDa FITC-dextran (*106*), ensuring the monolayer acts as a functional transport seal prior to blood perfusion. Concurrently, junctional maturation mapping is assessed via high-resolution immunofluorescence staining for VE-cadherin and CD31, verifying absolute cell-border continuity across the complex, printed dural curvatures (*107*). Last, real-time kinetic thrombosis quantification is functionally benchmarked using automated optical metrics to track continuous luminal occlusion velocity, time to clot, and relative fibrin-dense red thrombus volume under patient-specific stasis profiles.

Modeling the pathophysiology of the CW requires capturing two distinct disease phases. Because initial web development involves fibromuscular intimal hyperplasia rather than lipid-driven plaques, representing this structural phase requires cocultured bioprinted scaffolds with adjustable wall thicknesses to layer ECs over SMCs. Conversely, modeling the subsequent thromboembolic mechanism focuses primarily on regional hemodynamics. The intraluminal shelf induces localized fluid separation, WSS, and a high OSI. This causes blood stagnation and subsequent clotting behind the flap. Replicating this requires a confluent EC monolayer coating the vessel walls while leaving the web itself cell-free to mimic in vivo histology (*61*). This selective de-endothelialization is achieved via targeted laser ablation (>70% maximum output power) through the optically transparent hydrogel. The high resolution of light-based biofabrication permits precise replication of patient-specific web lengths (2.6-mm asymptomatic versus 3.5-mm symptomatic cohorts) (*35*) and insertion angles between the web and the posterior wall (*108*, *109*), which dictate the geometry of the recirculation zones. Capturing these millimeter-scale nuances is critical, as the projection length and angle into the carotid bulb fundamentally dictate localized fluid separation zones (*62*), and flow rates are tuned to match patient-specific shear rates derived from donor Doppler imaging. This allows for real-time tracking of clotting and embolization kinetics via fluorescence imaging and postassay scanning electron microscopy visualization.

Beyond hemodynamics, these platforms can evaluate the sex- and ethnicity-stratified hormonal variations linked to CW prevalence. Subjecting cocultured constructs to hormone-spiked media under continuous perfusion for up to 7 days enables direct observation of phenotypic cellular shifts. For instance, incorporating the 13 to 35% elevations in baseline estrogen activity and distinct age-related estradiol decline patterns observed in reproductive-aged African female cohorts (*48*) provides a mechanism to investigate the demographic disparities of the disease. Endothelial mechanosensors convert these coupled fluid and hormonal inputs into biochemical signals triggering a transition from a quiescent, atheroprotective state to a prothrombotic profile. In experimental models, these distinct phenotypic switches are conventionally quantified and verified via downstream molecular readouts, such as quantitative polymerase chain reaction for transcriptional shifts, Western blotting to assess protein expression and phosphorylation states, and semiquantitative immunofluorescence, flow cytometry, or enzyme-linked immunosorbent assays (ELISA) to isolate cell-surface marker presentation (*110*). In addition, live imaging of intracellular calcium signaling, cytoskeletal remodeling, and VE-cadherin clustering via genetically encoded indicators or fluorophore-conjugated antibodies has been used as an indirect way to detect EC activation from mechanical stimuli (*111*). The perfusate can be collected before perfusion and after cultivation to quantify changes in levels of inflammatory cytokines and thrombogenic markers such as vWF, tumor necrosis factor–α (TNF-α), and interleukin-6 (IL-6) (*112*).

### Current limitations in modeling

Despite the translational utility of these integrated validation pathways, several fundamental engineering and biological simplifications must be acknowledged. Structurally, the current framework relies on isolated, segmented regions of a patient’s neurovascular anatomy, such as a discrete portion of the superior sagittal sinus or the isolated carotid bifurcation bulb. Consequently, the present approach does not yet capture the full, continuous hierarchical vascular architecture of the cerebral drainage or arterial pathway. Replicating highly branched, multiscale networks on a single platform remains heavily restricted by asymmetric fluidic resistances and microchannel occlusion during manual cell-seeding phases. Biologically, the platform does not yet capture the broader cellular complexity of the cerebrovascular unit. In late-stage acute CVST, the clinical pathology transitions from simple intraluminal thrombosis to a multilineage parenchymal crisis. Localized fluid stagnation drives back-pressure that physically breaks down the blood-brain barrier, inducing severe cytotoxic and vasogenic edema in the surrounding cortex. Fully recapitulating these hemorrhagic and ischemic events in vitro requires the functional integration of the surrounding neural parenchyma, including astrocytes, pericytes, and neurons.

Similarly, modeling the true developmental pathogenesis of the CW remains limited by the microenvironmental demands of multilineage cocultures. Layering distinct, quiescent cellular boundaries, such as a confluent endothelial monolayer over a deep, fibroblast and SMC-dense wall matrix, requires complex, multistage media configurations and precise cross-linking profiles to prevent localized nutrient starvation and cellular phenotype drift.

Nevertheless, these light-based platforms provide an essential, iterative tool that bridges computational fluid modeling with experimental mechanobiology. By validating patient-specific CFD metrics against observable in vitro clotting behavior, this framework establishes a robust baseline for identifying the precise biophysical thresholds that govern rare and under-characterized cerebrovascular disorders.

## TRANSLATIONAL AND CLINICAL POTENTIAL

The translation of light-based bioprinting into actionable medicine relies on creating a distinct operational pipeline for venous and arterial pathologies. For CVST, translating patient-specific neurovascular geometries enables the dynamic assessment of stasis thresholds and individual hypercoagulability risk factors across complex dural structures. For CW, technology shifts toward simulating an aggressive arterial environment to evaluate localized antiplatelet response or mechanical intervention efficacy. In both regimes, the perfusion of whole blood under precise hemodynamic control allows quantitative analysis of fibrin polymerization, real-time microoptical occlusion tracking, and clot elasticity (*113*). Integrating these physical constructs with CFD establishes experimental-clinical feedback loops capable of defining patient-matched thrombotic risks and optimizing localized therapeutic regimens.

Artificial intelligence serves as a major accelerator for automating this personalized translation. When applied to patient cerebrovascular imaging, AI algorithms can identify subtle, high-risk morphological anomalies, such as variations in macroscale venous sinus branching for CVST or microscale carotid bifurcation shelf structures for CW. Combined with automated imaging segmentation pipelines, these data expedite the translation of patient scans into printable 3D files. Concurrently, data-driven optimization strategies refine printing parameters and bioink cross-linking densities, ensuring automated, highly consistent fabrication of complex, patient-matched vascular conduits (*114*).

The translational urgency varies between these two conditions due to current gaps in clinical data and diagnostic classification. While CVST management benefits from an established clinical framework, CW represents a condition of vital clinical importance with virtually no established mechanical or biological models. Personalized approaches are critically required to understand why specific CW patient cohorts remain highly refractory to conventional antithrombotic treatments. While invasive interventions like carotid endarterectomy or stenting achieve near-zero recurrence (*115*), fewer than 1% of patients with CW are selected for these procedures in clinical practice. This stark clinical gap stems directly from underdiagnosis and the frequent misclassification of CW as benign vascular variants or standard atherosclerosis on routine clinical imaging. Reconstructing these geometries using light-based bioprinting and microfluidic platforms directly resolves these diagnostic challenges.

Light-based lithography introduces a profound paradigm shift when evaluated against conventional in vitro platforms, such as 2D cultures, planar microfluidic devices, soft-lithography silicone blocks, and standard extrusion-based or sacrificial-casting models. Across the three critical axes of anatomical fidelity, hemodynamic control, and pathophysiological relevance, traditional platforms remain severely limited by rigid, simplified, or unphysiological geometries that introduce boundary-layer artifacts and nonnative fluid vectors. Conversely, DLP and VBP circumvent these fabrication constraints to seamlessly replicate nonuniform, multiscale, and patient-specific intracranial pathways. This structural precision authentically recapitulates both the macroscale low-shear stasis zones that govern venous sinus thrombosis propagation and the microscale high-shear flow separation gradients generated behind a CW shelf.

High-throughput fabrication can rapidly screen diverse patient cohorts, generating large, standardized datasets to populate AI-driven digital twins for experiment-less stroke prediction and clinical decision-making. There are existing studies that leverage printed patient vascular models to generate AI tools capable of personalized modeling and treatment evaluation that align with in vitro and in vivo observations (*65*). With the development of digital models, personalized thrombosis and stroke evaluation can become even more efficient. Such models require extensive real-world data to train on to ensure accuracy, and bioprinted vascular models represent a critical linchpin to bridging the gap between privacy-limited patient data AI training requirements. However, the specific translational readouts diverge by anatomy. In CVST models, high-fidelity replication of the macroscopic venous sinus junctions captures how global geometric features alter WSS gradients to promote stasis and red thrombus propagation. In contrast, CW models leverage the sub–50 μm resolution of DLP to model how submillimeter web variations, such as clinically identified averages of 2.6-mm asymptomatic versus 3.5-mm symptomatic webs, dictate local flow separation, high-shear platelet aggregation, and downstream microembolic shedding. The optical transparency of the hydrogel in VoC models allows one to visualize these distinct clot kinetics in real time, validating computational models and providing a reproducible, cellular-resolution platform to guide patient triage.

Figure 5 illustrates how in silico modeling of patient geometries can be translated to bioprinted vascular models for dynamic observations to be made. These results have the potential to represent in vivo phenomena, validated through correlation with patient records through retrospective or longitudinal studies.

**Fig. 5.**
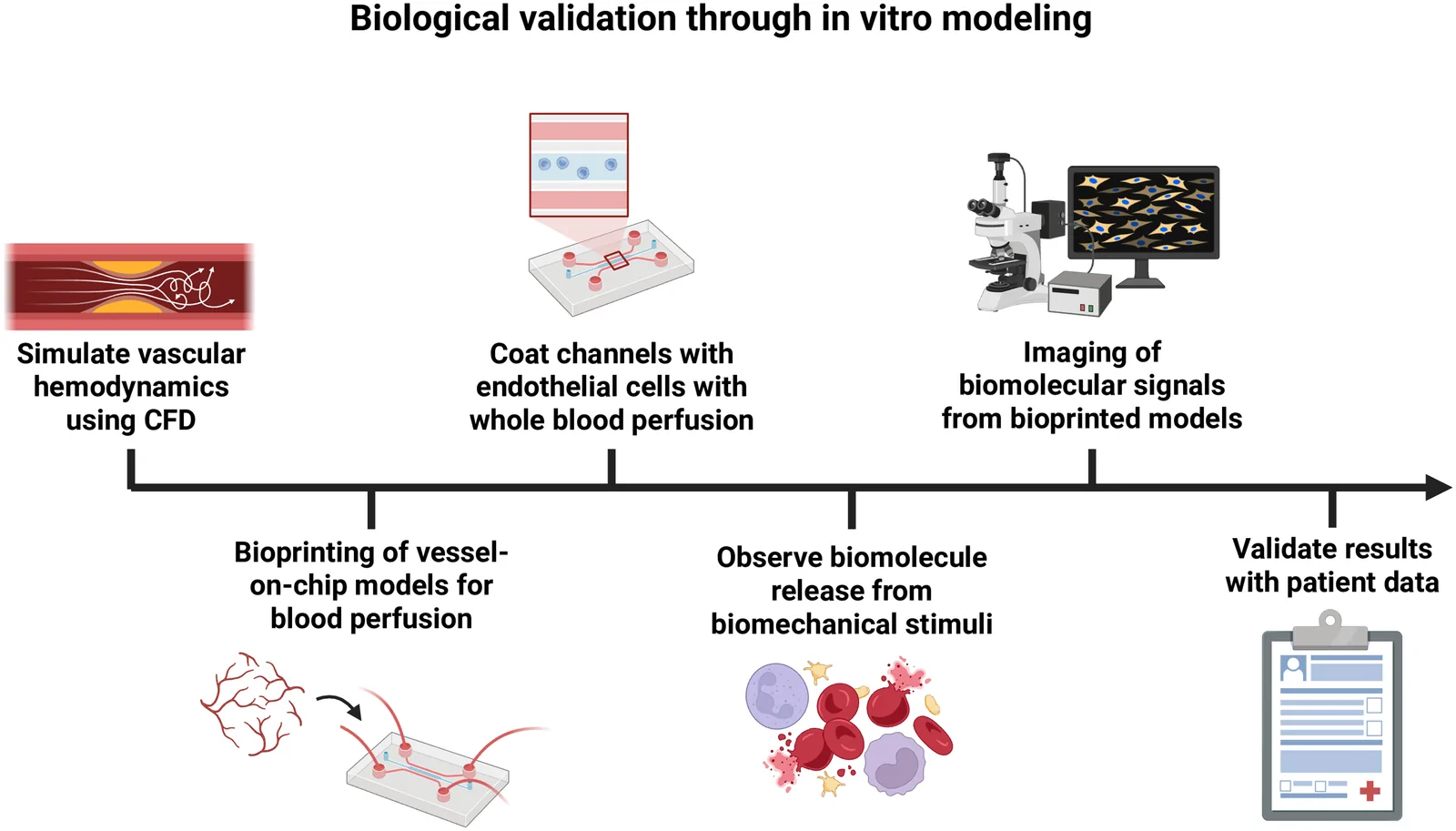
Bioprinting to clinical validation workflow. [Created in BioRender. Yap, N.A. (2026) https://BioRender.com/5kz4myt].

The primary translational advantage of this platform is its capacity to advance sex-specific vascular research and target women’s cardiovascular vulnerabilities. By introducing hormonal modulators like estrogen or progesterone into perfused, endothelialized networks, investigators can directly measure how specific hormone concentration amplitudes alter vascular responses. This capability bridges a critical translational gap, linking molecular endocrinology directly to physical vascular pathomechanics. Furthermore, with the U.S. FDA and EMA increasingly recognizing in vitro human cell–derived models as valid alternatives to conventional preclinical animal studies (*116*), this framework offers a scalable, ethically sustainable pathway for investigating rare or underrepresented female vascular conditions that lack sufficient animal correlates.

To fully realize the predictive potential of these models, important directions for future work must transition these hydrodynamic platforms into fully integrated biological systems. While current implementations excel at capturing acute biophysical and fluid-dynamic vectors, next-generation platforms must explicitly map the chronic tissue-level variables driving these diseases. Specifically, future iterations must integrate multilineage inflammatory signaling cascades within the vascular wall to study the fibromuscular progression of intimal hyperplasia in CW development. In addition, transitioning from generic cell lines and healthy blood donors to assays using patient-derived hypercoagulable whole blood, specifically isolating active postpartum states, inherited thrombophilias, or oral contraceptive-induced hematological changes, remains an essential frontier. Resolving these complex cellular, hormonal, and hematological horizons represents the next crucial milestone in establishing truly predictive, patient-specific platforms for female cerebrovascular diseases.

## CONCLUSION AND CLOSING REMARKS

Advancing the modeling of neurovascular thrombosis demands more than anatomical replication. It requires an integrated engineering strategy that captures the full interplay of geometry, flow, and biology. Light-based bioprinting, supported by machine learning–driven segmentation, delivers this capability. Its optical precision, material control, and scalability allow the faithful reconstruction of cerebral venous sinuses and CWs, transforming how thrombosis is studied and understood. These bioprinted systems overcome the structural and optical constraints of conventional platforms, enabling direct observation of shear forces, endothelial activation, and clot evolution under physiologically relevant flow. By bridging mechanobiology and fabrication, they validate the principles of Virchow’s triad within living, patient-specific architectures. This technological shift marks a decisive step toward translational relevance. It establishes light-based bioprinting as the foundation for building reproducible, ethical, and human-centered vascular models that can guide therapeutic testing, surgical planning, and risk prediction. More profoundly, it provides a long-overdue framework for advancing women’s vascular health, ensuring that innovations in cardiovascular medicine are both precise and inclusive.

While light-based bioprinting offers substantial advantages, several experimental challenges must be rigorously managed to ensure model fidelity. High cell densities within bioinks lead to optical scattering, which can compromise print resolution and curing depth during VBP. Postprint processing must account for bioink swelling, which alters the carefully designed luminal dimensions. Furthermore, integrating soft, highly compliant biomaterials with rigid fluidic connectors frequently causes interface leakage during perfusion.

In downstream whole-blood thrombosis assays, controlling donor variability, standardizing recalcification protocols for anticoagulant reversal, and managing platelet function drift over time are critical. In addition, the choice of cellular source, whether utilizing primary venous ECs, generic HUVECs, or patient-derived iPSC-ECs, will profoundly impact the biological accuracy of the construct, particularly regarding shear responsiveness.
